# Genomewide mechanisms of chronological longevity by dietary restriction in budding yeast

**DOI:** 10.1111/acel.12749

**Published:** 2018-03-25

**Authors:** Sergio E. Campos, J. Abraham Avelar‐Rivas, Erika Garay, Alejandro Juárez‐Reyes, Alexander DeLuna

**Affiliations:** ^1^ Unidad de Genómica Avanzada (Langebio) Centro de Investigación y de Estudios Avanzados del IPN Irapuato Guanajuato Mexico; ^2^ Present address: Department of Developmental and Molecular Biology Albert Einstein College of Medicine Bronx NY USA

**Keywords:** aging, cell cycle, cell cycle arrest, chronological lifespan, dietary restriction, genomewide profiling, *Saccharomyces cerevisiae*, Ste12, transcription factors

## Abstract

Dietary restriction is arguably the most promising nonpharmacological intervention to extend human life and health span. Yet, only few genetic regulators mediating the cellular response to dietary restriction are known, and the question remains which other regulatory factors are involved. Here, we measured at the genomewide level the chronological lifespan of *Saccharomyces cerevisiae* gene deletion strains under two nitrogen source regimens, glutamine (nonrestricted) and γ‐aminobutyric acid (restricted). We identified 473 mutants with diminished or enhanced extension of lifespan. Functional analysis of such dietary restriction genes revealed novel processes underlying longevity by the nitrogen source quality, which also allowed us to generate a prioritized catalogue of transcription factors orchestrating the dietary restriction response. Importantly, deletions of transcription factors Msn2, Msn4, Snf6, Tec1, and Ste12 resulted in diminished lifespan extension and defects in cell cycle arrest upon nutrient starvation, suggesting that regulation of the cell cycle is a major mechanism of chronological longevity. We further show that *STE12* overexpression is enough to extend lifespan, linking the pheromone/invasive growth pathway with cell survivorship. Our global picture of the genetic players of longevity by dietary restriction highlights intricate regulatory cross‐talks in aging cells.

## 1 INTRODUCTION

Dietary restriction—a reduction in calorie intake without malnutrition, or substitution of the preferred carbon or nitrogen source—extends lifespan in virtually all species studied in the laboratory (Mair & Dillin, 2008). Dietary restriction has been associated with protection against age‐associated disease in mice, including neurodegenerative disorders (Zhu, Guo & Mattson, 1999) and cancer (Yamaza et al., 2010), promoting longer lifespan and healthier aging (Fontana & Partridge, 2015). Importantly, this intervention reduces the mortality rate in nonhuman primates (Colman et al., 2014) and delays the onset of aging‐related physiological changes in humans (Holloszy & Fontana, 2007), making dietary restriction the most promising intervention targeted to extend human lifespan. Yet, we are still missing a global picture of the genetic architecture of such lifespan response, which is needed to grant a deeper understanding of the genotype–phenotype relationship of aging and longevity (Schleit et al., 2013).

The budding yeast *Saccharomyces cerevisiae* has been a pivotal model organism in the discovery of the molecular basis of aging. Two aging models are widely used in this organism: the replicative lifespan of yeast, which refers to the number of times a single yeast cell can divide, and the chronological lifespan (CLS)—a measure of the viability of a population during stationary phase through time. The latter provides a good model for inquiring the molecular changes faced by postmitotic cells (Longo, Shadel, Kaeberlein & Kennedy, 2012).

In yeast, dietary restriction results in lifespan extension at least through the modulation of the conserved TOR and Ras/cAMP/PKA pathways, which regulate cellular growth and maintenance in response to nutrient availability (Kaeberlein et al., 2005). Depletion of TOR components, Tor1 and Sch9, results in CLS extension (Powers, Kaeberlein, Caldwell, Kennedy & Fields, 2006). Under low nutrient conditions, the serine/threonine kinase Rim15 phosphorylates transcription factors Msn2, Msn4, or Gis1, activating a maintenance response (Fabrizio, Pletcher, Minois, Vaupel & Longo, 2004). However, the *msn2*Δ*msn4*Δ*gis1*Δ triple mutant still shows lifespan extension by DR (Wei et al., 2008). Moreover, transcriptomic evidence and in silico predictions suggest that a larger number of up‐ and downstream genes are involved in lifespan extension (Choi et al., 2017; Wuttke et al., 2012); most of these candidates lack direct phenotype confirmation. These observations suggest that there is an unknown number of longevity regulators that are yet to be identified.

Aging research in yeast has recently taken advantage of genomewide approaches, enabling a comprehensive description of genes involved in lifespan regulation. For instance, a recent systematic study of replicative lifespan of most viable deletion strains revealed that translation, the SAGA complex, and the TCA cycle mediate longevity (McCormick et al., 2015). Several studies have aimed to estimate the stationary‐phase survival of single deletion mutants in parallel (Fabrizio et al., 2010; Garay et al., 2014; Gresham et al., 2011; Matecic et al., 2010; Powers et al., 2006), showing that autophagy, vacuolar protein sorting, regulation of translation, purine metabolism, chromatin remodeling, and the SWR1 complex are major determinants of longevity. However, we are still missing a direct comparison of the lifespan effects of such gene deletions under nutrient‐rich and restricted conditions that would allow to systematically address the mechanisms of longevity by dietary restriction.

The aim of this study was to generate a global picture of the underlying genetics of lifespan extension by dietary restriction, by systematically describing gene–diet interactions in yeast. Specifically, we compared the CLS of a collection of 3,718 knockout mutants aged in media with glutamine (nonrestricted) or γ‐aminobutyric acid (GABA, dietary‐restricted) nitrogen source, for which we used a high‐resolution parallel phenotyping assay (Garay et al., 2014). These screens revealed that at least 473 genes have a role in lifespan extension by GABA. Subsequent analyses uncovered the major biological processes involved and a comprehensive catalogue of transcription factors that control lifespan extension in this mode of dietary restriction. Many gene deletions of such regulators were defective in arresting the cell cycle upon nitrogen or carbon starvation, suggesting that cell cycle control is a mechanism of chronological longevity. We focus on the transcription factor Ste12 and discuss upstream signaling pathways and downstream processes that could underlie its pro‐longevity and cell cycle roles in response to nutrients.

## 2 RESULTS

### 2.1 Genomewide profiling of yeast chronological lifespan under different dietary regimens

To identify gene deletions that modify the effect of dietary restriction, we set out to establish a model of lifespan extension in response to nutrient limitation. In yeast, lifespan is extended either by limiting the concentration of glucose in the medium or using a nonpreferred source of nitrogen (Jiang, Jaruga, Repnevskaya & Jazwinski, 2000; Powers et al., 2006). We measured the lifespan of a wild‐type (WT) strain aged under different nitrogen sources by adapting an established method for quantitative analysis of yeast CLS based on outgrowth kinetics (Murakami, Burtner, Kennedy & Kaeberlein, 2008) to a high‐throughput robotic platform (see Section 4). The half‐life of the WT strain varied substantially, from 5.6 days in the rich nitrogen source ammonium to 26.5 days in GABA (Table 1; Fig. S1a). The use of GABA as the sole nitrogen source resulted in extended lifespan without major effects on the growth kinetics (Table 1; Fig. S1b); therefore, we selected GABA as our dietary‐restricted condition. We used glutamine in the nonrestricted medium, as it resulted in one of the shortest half‐lives measured and it is a preferred nitrogen source (Godard et al., 2007); ammonium sulfate was not used because of its toxicity in stationary phase (Santos, Leitão‐correia, Sousa & Leão, 2015). After repeating the experiment only under the selected conditions, we observed that our nitrogen‐based dietary restriction model resulted in a 91% extension of the CLS, from a WT half‐life of 14.7 days in glutamine to 28.1 days in GABA (Fig. S1c).

**Table 1:** Chronological lifespan of the WT strain aged under different nitrogen sources

Nitrogen source	Death rate, *r* a	Half‐life, days^a^	Doubling time, hours^b^
Ammonium	−0.137 ± 0.010	5.6 ± 0.4	2.70 ± 0.11
Methionine	−0.061 ± 0.005	11.4 ± 0.9	3.59 ± 0.09
Glutamine	−0.057 ± 0.004	13.1 ± 1.0	2.64 ± 0.09
Asparagine	−0.049 ± 0.004	15.3 ± 1.0	2.69 ± 0.09
Phenylalanine	−0.046 ± 0.006	15.9 ± 1.8	2.91 ± 0.12
Leucine	−0.041 ± 0.003	18.0 ± 1.3	4.31 ± 0.02
Isoleucine	−0.041 ± 0.004	18.3 ± 2.2	3.05 ± 0.12
Valine	−0.039 ± 0.004	18.9 ± 2.1	2.76 ± 0.07
GABA	−0.029 ± 0.005	26.5 ± 4.4	2.82 ± 0.07

a Death rate and half‐life are obtained from fitting survival curves to an exponential decay model; values are the average ± *SEM* from at least five experiments.

b Obtained from the rate during the exponential phase of growth.

**Figure 1:**
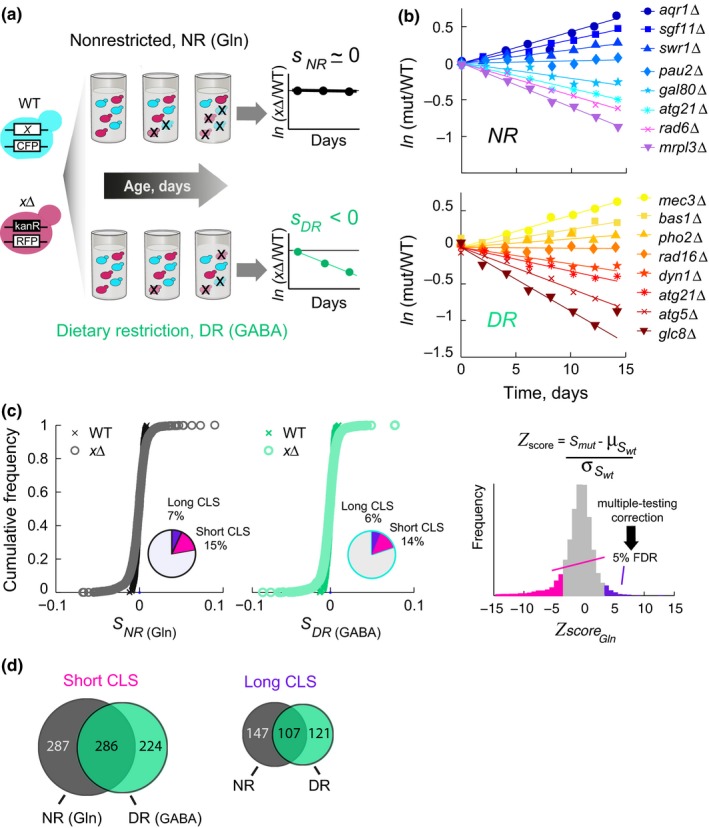
Genomewide profiling of chronological lifespan under two dietary regimens. (a) Schematic representation of the screening strategy; an RFP‐labeled deletion strain (*x*Δ) is aged in co‐culture with a CFP‐labeled WT strain; a survival coefficient (*s*) relative to WT is obtained for each mutant under each aging condition. (b) Examples of the survival coefficients obtained; selected long‐lived and short‐lived mutants under glutamine (nonrestricted, upper panel) and GABA (dietary restriction, lower panel) are shown. For comparison purposes, initial offsets were corrected to start at zero. (c) Cumulative distributions of *s* values for the 3,718 single deletion strains under glutamine (gray circles) and GABA media (green circles), 264 WT replicas were included (black and green crosses). Right, *Z*‐scores were obtained (only glutamine is shown) by comparing the mutants’ survival coefficient (*s*) to the *s* distribution of WT reference experiments; the multiple‐testing correction was used to define hits at a 5% FDR. (d) Venn diagrams show the overlapping short‐ and long‐lived mutant strains scored under glutamine (NR, dark gray) and GABA (DR, green)

To identify the genetic determinants of lifespan extension by dietary restriction at the genomewide level, we measured the CLS of 3,718 gene‐knockout strains under GABA (dietary‐restricted) and glutamine (nonrestricted) media. We used a profiling assay based on the measurement of a relative survival coefficient (*s*) of each knockout strain aged in co‐culture with the WT strain (Figure 1a). Our model of stationary‐phase survivorship assumes that yeast cells die in an exponential manner, and thus, the natural logarithm of the ratio of each mutant strain and the WT reference was adjusted to a linear fit; short‐lived mutants had negative slopes (survival coefficient, *s*), while long‐lived strains had positive slopes (see examples under the two conditions in Figure 1b). In addition, we obtained the *s* of 264 WT versus WT competitions under both nutrimental conditions and used the mean and standard deviation of this population to calculate a Z‐score for each mutant, assuming that the variance in the mutants was the same as in the WT. Finally, we used the Benjamini–Hochberg false‐discovery rate (FDR) to correct for multiple hypothesis testing (Figure 1c). Using an FDR threshold of 5%, we scored 573 significantly short‐lived and 254 long‐lived single knockout strains in the nonrestricted medium, while dietary restriction resulted in 510 short‐lived and 228 long‐lived mutants (Data S1). We confirmed that most mutants showed a good fit to such exponential decay model, and observed no significant differences between the distribution of errors in the fit among the different phenotypic categories (*p *>* *.05 Wilcoxon rank‐sum test; Fig. S2).

A considerable fraction of the strains tested showed condition‐specific CLS effects (Figure 1d). Most condition‐specific effects were associated to coherent phenotypes (short or long‐lived under both conditions) or neutral phenotypes under one of the conditions: Only seven gene deletions were long‐lived under glutamine showing the opposite effects under GABA, while eleven gene deletions were short‐lived under glutamine but long‐lived under GABA.

To validate our genomewide screens, we turned again to the small‐scale outgrowth‐kinetics CLS approach (Murakami et al., 2008) and measured in single‐strain cultures the lifespan of a set of randomly selected knockouts with significant lifespan effects (5% FDR). Twelve of 16 (75%) strains that were re‐tested under nonrestricted glutamine medium recapitulated the CLS effects observed in the genomewide screen (Fig. S3a; *p *<* *.05, *T* test), while 11 of 17 (65%) strains were consistent with the results under GABA (Fig. S3b). Notably, the rate of validation is considerably higher compared to screening assays that have used pooled yeast deletion strains (6–31% hits validated) (Fabrizio et al., 2010; Matecic et al., 2010). To directly compare the results obtained with the conventional single culture CLS assay and with our high‐throughput screen, we modeled a survival curve from the survival coefficients of the corresponding knockout strains (Fig. S3a, b). Together, the results of these validation experiments indicate that our profiling approach provides accurate and quantitative CLS scores under different conditions, allowing the description of gene–diet interactions.

### 2.2 Systematic identification of dietary restriction genes in yeast

**Figure 2:**
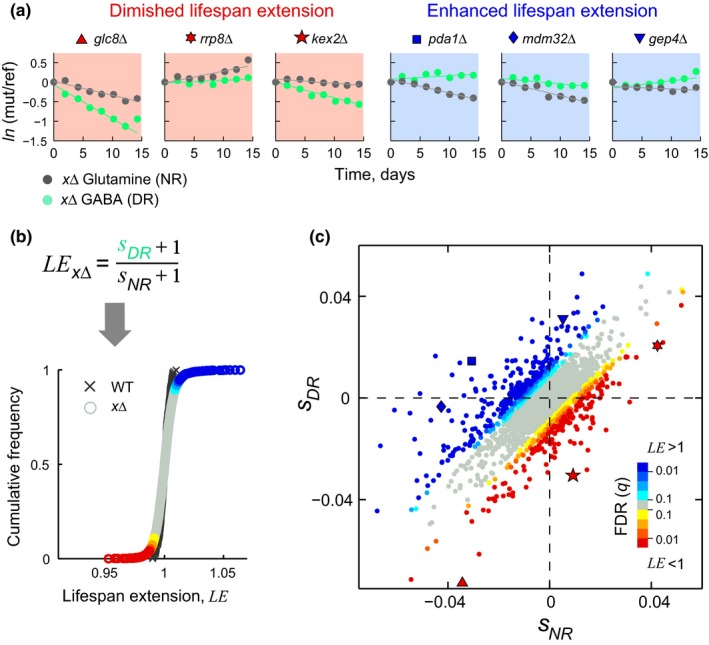
A comprehensive compendium of dietary restriction genes. (a) Selected knockout strains with differences in their selection coefficients obtained under glutamine (nonrestricted) and GABA (dietary restriction), resulting in diminished (red panels) or enhanced (blue panels) lifespan extension. (b) A relative lifespan extension, *LE*, was calculated for each mutant strain (circles) and compared to that of the WT replicates (crosses) to score statistical significance. (c) Scatter plot of the survival coefficients of 3,718 deletion strains under nonrestricted medium (horizontal axis) and dietary restriction (vertical axis); data points above or below the diagonal are colored according to the lifespan extension (*LE*) significance (*q*). Data points shown with special symbols are the selected examples in panel (a) of significantly diminished (red) or enhanced (blue) lifespan extension

To gain insight into the genes that mediate the lifespan‐extending effects of dietary restriction, we searched for deletion strains that showed differential relative CLS effects under the two nitrogen sources. Specifically, we sought to identify strains that showed a shorter survival coefficient under GABA compared to the nonrestricted glutamine medium (diminished lifespan extension); likewise, we scored cases where the survival coefficient was relatively larger under GABA (enhanced lifespan extension) (see examples in Figure 2a).

We quantitatively defined the relative lifespan extension of each knockout as $LE=\frac{s_{\mathrm{DR}}+1}{s_{\mathrm{NR}}+1}$ (see Section 4). Next, we compared the *LE* of each deletion strain to the distribution of *LE* in 264 independent WT replicates to obtain a *Z*‐score (Figure 2b). After correcting for multiple tests, we scored 583 mutant strains with a significant *LE*; for higher stringency, we filtered out strains that did not show a significant CLS effect in either condition. The final list included 473 gene‐knockouts with altered dietary restriction response (5% FDR; Figure 2c; Data S2). This comprehensive set, which we termed DR genes, includes 220 knockouts with diminished longevity (*LE* < 1) and 253 gene‐knockouts that displayed enhanced lifespan extension (*LE* > 1). These results indicate that many gene–environment interactions underlie longevity by nitrogen restriction in yeast.

In addition to their diminished or enhanced phenotypes based on *LE*, we classified DR genes based on their effects under glutamine and GABA (Data S2). This categorization was useful to identify genes of particular cellular functions that behaved in a similar manner. For instance, DR gene deletions with diminished longevity (*LE* < 1) that were long‐lived under both glutamine and GABA were enriched for ribosome biogenesis (5% FDR), while different functions related to autophagy and protein‐targeting to vacuole were common in strains with diminished longevity and short‐lived phenotypes under both conditions. On the other hand, genes of mitochondrial translation were enriched in deletions with enhanced longevity and short‐lived phenotypes under both glutamine and GABA (Data S2).

To estimate the sensitivity of the identified DR genes to the mode of dietary restriction tested, specifically the source of nitrogen, we repeated our analysis using a different nonrestricted medium reference. Specifically, we used data from a previously reported genomewide screen performed in 2% glucose SC medium with ammonium sulfate as the sole nitrogen source (Garay et al., 2014), to obtain a new relative lifespan extension value for each knockout, *LE*_GABA/NH4_. We observed a strong skew in the distribution of *LE*_GABA/NH4_ values, for both sets DR genes with diminished‐*LE* (*p* < 10^−28^, one‐tailed Wilcoxon ranked‐sum test) or enhanced‐*LE* (*p* < 10^−34^) using the GABA/glutamine comparison (Fig. S4a). Nonetheless, a number of *LE* values did show a strong dependency on the nitrogen source used in the analysis.

To look into possible processes underlying media‐specific effects on lifespan extension, we focused on the top 20 genes with the highest absolute difference in the *LE* values (Fig. S4b). Eleven of 20 such genes were located to the mitochondria (YeastMine, Saccharomyces Genome Database), but not significant enrichment to any GO term was found (Fig. S4c). In this regard, the screen performed in ammonium was carried out in a buffered medium (Garay et al., 2014), while the ones presented here are not, implying that some of the condition‐specific effects could be due to different pH in the stationary‐phase cultures. In conclusion, these comparative analyses suggest that, while some gene deletions have diminished or enhanced lifespan extension only under specific conditions, most DR genes influence the lifespan response to nutrient limitation regardless of the nitrogen source used to model dietary restriction.

### 2.3 Functional classification of dietary restriction genes

**Figure 3:**
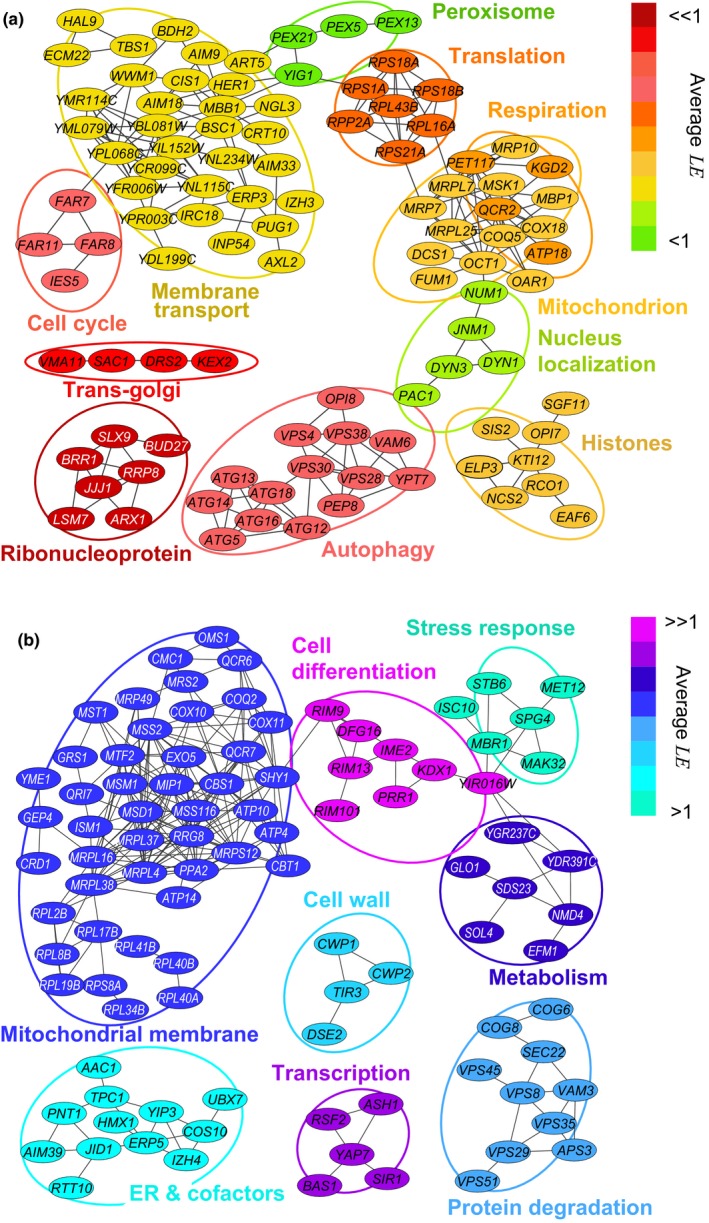
Functional clustering of dietary restriction genes. Network representation of functional clusters of genes with (a) diminished (*LE* < 1) and (b) enhanced (*LE *> 1) lifespan extension. Edges denote agreement between pair of genes (κ > 0.35), which suggests functional association between the genes; clusters were formed between at least four associated genes. Node color code indicates decreasing or increasing ranks of mean *LE* for each cluster

To systematically describe which downstream cellular functions influence lifespan extension by dietary restriction under the specific nitrogen conditions tested, we sought to classify the 473 DR genes according to their annotated functional features. We used a kappa statistic approach (Huang, Sherman & Lempicki, 2009) to cluster genes by shared GO terms and mutant phenotypes—as reported in the Saccharomyces Genome Database—providing associations between the gene deletions based on their phenotypes and other genes’ features reported. With this approach, genes were clustered into discrete groups even when there is no common GO association among them, allowing a nonsupervised identification of the cellular processes represented. The analysis was performed separately for genes with diminished (*LE* < 1) or enhanced (*LE* > 1) lifespan extension (Figure 3; Data S3).

Some clusters recapitulated cellular functions previously related to lifespan regulation, such as autophagy (Meléndez et al., 2003), mitochondrial function (Aerts et al., 2009), and cytosolic translation (Hansen et al., 2007). Importantly, this classification also revealed novel processes such as maintenance of cell cycle arrest mediated by pheromone and establishment of nucleus localization in the cell. Hence, our screen re‐discovered processes previously known to influence the dietary response and, at the same time, it allowed the identification of novel genes and processes related to longevity by dietary restriction.

Deletion of genes necessary for the maintenance of cell cycle arrest resulted in a diminished lifespan extension. Specifically, deletion of pheromone‐responsive genes *FAR7* and *FAR8* had short‐lived phenotype under dietary restriction (Figure 3a). In yeast, these Far proteins prevent cell cycle recovery after pheromone exposure, possibly by inhibiting *CLN1*‐3 (Kemp & Sprague, 2003). Moreover, mutations in genes required for processing and correct localization of ribonucleoprotein complexes resulted in a strongly diminished lifespan extension. While it is well established that ribosomal function is downregulated in response to TOR inhibition or nutrient depletion (Hansen et al., 2007), our screen pointed to specific proteins involved in pre‐rRNA processing (Slx9), nucleolar rRNA methyltransferase (Rrp8), nuclear export of pre‐ribosomal subunits (Arx1), and translational initiation (Bud27). Likewise, deletion of *DYN1*‐*3*,* JNM1*,* NUM1*, and *PAC1*, all involved in nuclear movement along microtubules, resulted in diminished lifespan extension. In this regard, it is known that certain cellular processes needed for extended longevity, such as autophagy, require intact function of microtubules (Köchl, Hu, Chan & Tooze, 2006). However, the relationship between nuclear localization and lifespan extension remains unexplored.

We also found clusters with enhanced lifespan extension, such as mitochondrial function (Figure 3b). Dietary restriction shifts metabolism toward respiration (Lin et al., 2002) but, at the same time, impaired respiration can promote longevity in yeast and nematodes through enhanced retrograde response and activation of anaplerotic pathways (Cristina, Cary, Lunceford, Clarke & Kenyon, 2009). Although we did not follow up on this intriguing observation, we speculate that a higher demand for respiration during dietary restriction could lead to the activation of compensatory pathways. Other feedback mechanisms may account for the alleviation of deleterious effects observed in other deletion strains: For example, the short‐lived phenotypes of deletion of cell‐wall genes *CWP1, DSE2,* and *TIR3* were largely alleviated under dietary restriction. Taken together, these findings suggest that lifespan extension in response to dietary restriction is a complex phenotype resulting from the interplay of many downstream cellular processes.

Importantly, we carried out an experiment to rule out the possibility that particular groups of DR genes were scored due to function‐specific alterations in the fluorescence signal that was used to measure the CLS of knockout strains (e.g., accumulation of fluorescent proteins in autophagy‐deficient mutants). To this end, we chose several autophagy and ribosomal protein genes, along with a set of randomly selected DR genes (20 knockouts altogether). At different time points in stationary phase, we measured single‐cell fluorescence signal of the selected strains using flow cytometry (see Section 4). The average signal increased slightly in the WT, and most mutant strains tested showed a very similar trend (Figure S5a–b). A number of strains did show differences in initial signal or signal change compared to the WT, including *vps51*Δ and *atg13*Δ (two of five autophagy and vacuolar protein sorting mutants tested). However, we observed no correlation between signal changes and the measured survival coefficients (*p *>* *.05, Pearson correlation; Figure S5b), suggesting that specific fluorescence signal dynamics did not introduce a systematic bias in our survival results.

### 2.4 A defined set of transcription factors controlling lifespan extension by dietary restriction

Complex phenotypic responses are frequently coordinated by transcriptional regulation of functionally related genes. To investigate the transcriptional regulation of longevity by dietary restriction, we analyzed our set of DR genes using an algorithm to search for the transcriptional regulators of these genes (Table 2). Specifically, we used TFRank (Gonçalves et al., 2011), a graph‐based approach that takes advantage of all known regulatory paths in *S. cerevisiae*. A weight is assigned to each TF according to the presence or absence of target genes and their regulators in the regulatory network of *S*. *cerevisiae*. The weight of the transcription factor increases according to the number of direct and indirect targets to rank the transcription factors; this weight is re‐evaluated through a diffusion coefficient, which takes into account the number of layers of the transcription factors hierarchy to obtain a list of prioritized regulatory players. Based on this analysis, we established a possible role in lifespan extension to the top‐ranked transcription factors, which included regulators that were not in our gene‐knockout screen due to gene essentiality or sterility. Transcription factors within the top 5% ranked DR regulators included Msn2 and Msn4; these transcription factors are well‐known regulators of lifespan (Fabrizio et al., 2004). Intriguingly, most top‐ranked transcription factors had not been previously associated with longevity in yeast. Noteworthy, the top hits (Ace2, Ash1, Tec1, Spf1, and Ste12) regulate different aspects of cell cycle progression.

**Table 2:** A catalogue of transcription factors regulating dietary restriction genes in yeast (DR regulators, top 5% rank)

Rank	TF	Weight^a^	% Regulated^b^	Description
1	Ace2	3.15	75.5	Involved in G1/S transition of the mitotic cell cycle; activates cytokinetic cell separation
2	Ash1	2.94	50.4	Negatively regulates G1/S transition of mitotic cell cycle; activates pseudohyphal growth
3	Tec1	2.81	63.0	Transcription factor targeting pseudohyphal growth genes and Ty1 expression
4	Sfp1	2.55	66.3	Regulates ribosomal protein genes, response to nutrients and stress, and G2/M transitions of the mitotic cell cycle
5	Ste12	2.45	58.2	Activates genes involved in mating or pseudohyphal growth pathways
6	Bas1	2.36	44.9	Involved in regulating the expression of genes of purine and histidine biosynthesis
7	Snf6	2.19	37.4	Subunit of the SWI/SNF chromatin remodeling complex
8	Msn2	2.10	54.3	Regulation of transcription in response to a wide variety of stresses
9	Yrm1	1.79	44.5	Transcription factor involved in multidrug resistance
10	Gcn4	1.76	46.7	Activator of amino acid biosynthetic genes; responds to amino acid starvation
11	Ixr1	1.73	26.4	Transcriptional repressor that regulates hypoxic genes during normoxia
12	Abf1	1.723	45.3	DNA‐binding protein with possible chromatin‐reorganizing activity
13	Msn4	1.65	44.0	Regulation of transcription in response to a wide variety of stresses
14	Rap1	1.53	41.4	Essential DNA‐binding transcription regulator; role in chromatin silencing and telomere length

a TFRank weight (Gonçalves et al., 2011).

b Percentage of dietary restriction genes regulated by the transcription factor.

**Figure 4:**
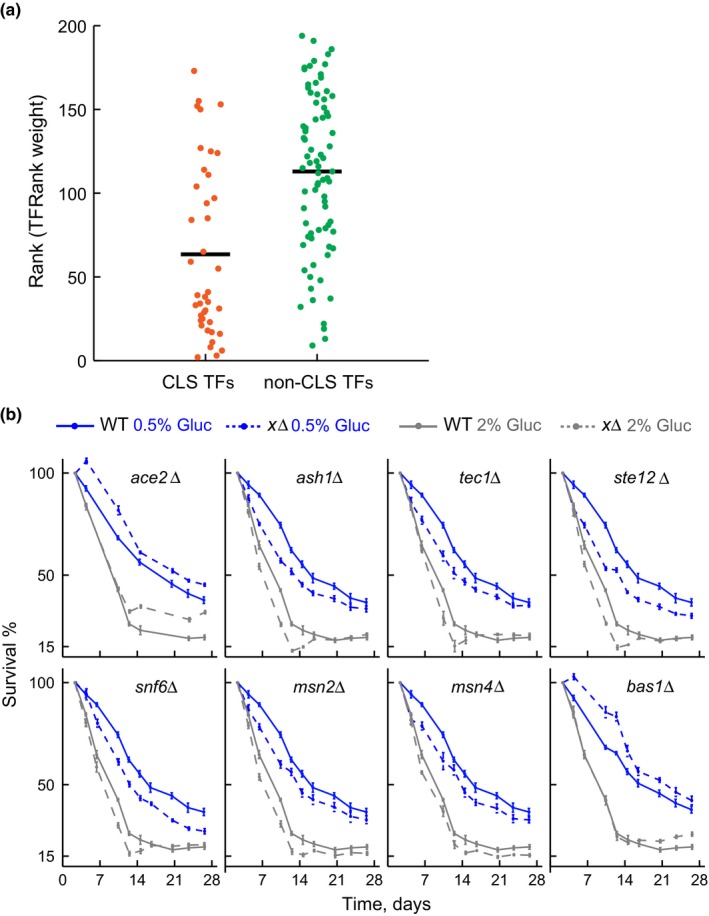
Altered chronological lifespan in strains deleted for dietary restriction regulators. (a) Plots indicate the rank of transcription factors (TFRank analysis of DR genes, *n *=* *197) with a gene deletion effect (CLS TFs, *n *=* *40) and without an effect (non‐CLS,* n *=* *79 of 119); only 119 transcription factors measured under both dietary conditions are considered. Solid black lines indicate the average rank of each group. (b) Survival curves of WT and gene deletion strains aged in SC medium with 2% glucose (nonrestricted, gray lines) or 0.5% glucose (dietary restriction, blue lines). Deletion strains (discontinuous lines) are for genes coding for transcription factors Ace2, Ash1, Tec1, Ste12, Snf6, Msn2, Msn4, and Bas1. Error bars are the *SEM* (*n *=* *5)

To assess the predictive power of the TFRank approach, we compared the ranks of the transcription factors that showed gene deletion lifespan phenotypes either in glutamine or GABA media (CLS transcription factors) with that of transcription factors showing no effect on lifespan. DR regulators with a deletion phenotype were typically ranked higher by the TFRank algorithm than regulators with no CLS effect (*p *< 10^−5^, Wilcoxon ranked‐sum test) (Figure 4a). The average ranks for transcription factors were 63.5 and 112.9 for regulators with and without a CLS gene deletion phenotype, respectively. This indicates that the downstream DR genes and cellular pathways identified in our genomewide screen are phenotypically linked to a coherent set of transcription factors.

The set of DR genes was defined from our genome screens under a particular dietary restriction regime based on nitrogen limitation. We thus decided to evaluate the role of those DR regulators in lifespan extension by calorie restriction, a classic form of dietary restriction in yeast and other organisms (Mair & Dillin, 2008). Specifically, we generated de novo deletion mutants for eight top‐ranked transcription factors and measured their CLS in 2% and 0.5% glucose using the small‐scale outgrowth‐kinetics approach. Strikingly, all transcription factor gene deletions resulted in altered CLS under 0.5% glucose: Six strains were short‐lived, while the remainder strains (*ace2*Δ and *bas1*Δ) were long‐lived under the glucose‐restricted condition (Figure 4b). As expected, the same mutants showed altered CLS in SC medium with glutamine or GABA as nitrogen source (Fig. S6). Most of the observed lifespan effects under glucose‐ or nitrogen‐restricted conditions were moderate, which is in agreement with the fact that transcription factors in yeast typically act upon overlapping targets, providing functional compensation to one another (Zheng et al., 2010). Together, these results show that top TFRank hits are general regulators of lifespan extension by dietary restriction in yeast.

### 2.5 Many of the transcription factors involved in lifespan regulation mediate cell cycle arrest in response to nitrogen or carbon starvation

**Figure 5:**
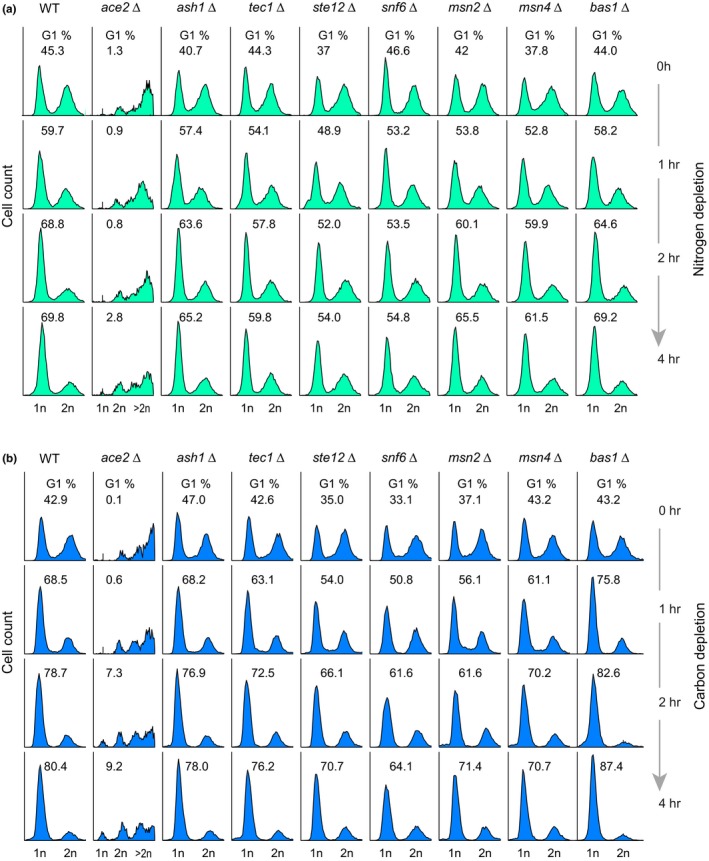
Several dietary restriction regulators mediate cell cycle arrest in response to nutrient limitation. Histograms show the DNA content of populations of WT and transcription factor mutant cells at 0, 1, 2, and 4 hr after nitrogen (a) or carbon (b) depletion. Cellular DNA content was measured by SYTOX‐Green staining followed by flow cytometry. Numbers indicate the percentage of cells at G1 in each population

Our functional classification of DR genes and TFRank analysis underscored the contribution of cell cycle control as a mechanism of lifespan regulation. To further explore the association between longevity by dietary restriction and the cell cycle, we directly measured the cell cycle status of DR regulator mutants after nutrient depletion. Specifically, we measured the DNA content of growing cells starved for nitrogen or carbon by flow cytometry. Interestingly, most mutants (Tec1, Ste12, Snf6, Msn2, and Msn4) failed to arrest efficiently in G1 after nitrogen or carbon depletion (Figure 5a, b). The long‐lived *bas1*Δ cells arrested faster compared to the WT, but only under carbon starvation (Figure 5b). The *ace2∆* strain analysis showed events of large DNA content; this mutation affects mother–daughter cell separation, which explains the large fraction of events with >2n DNA. These findings suggest that many of the transcription factors that act as DR regulators are involved in the cell cycle control or possibly polarized growth in response to nutrient limitation.

### 2.6 Ste12 is a positive regulator of longevity by dietary restriction and cell cycle arrest in response to nutrients

**Figure 6:**
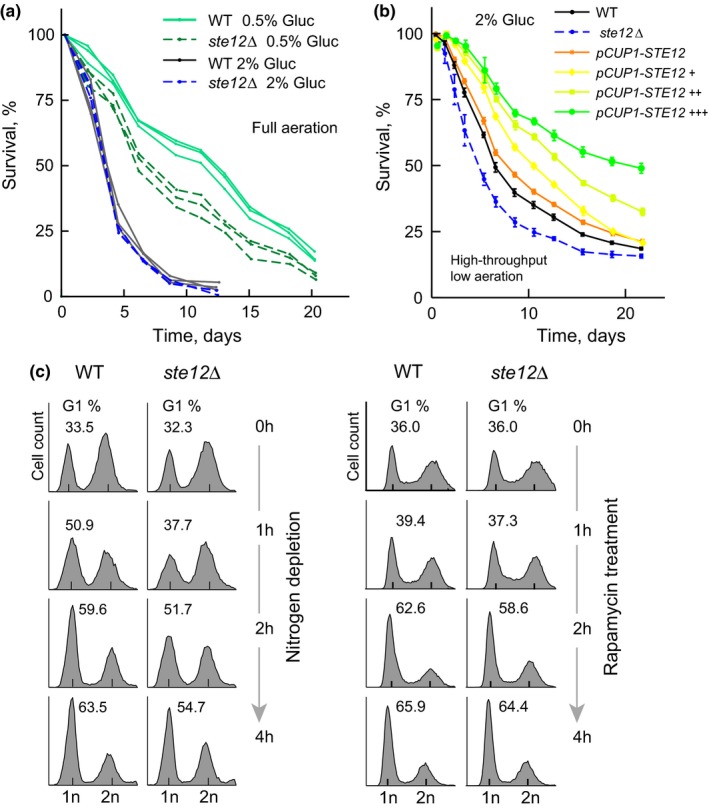
Ste12 is a positive regulator of longevity by dietary restriction. (a) Survival curves of WT and *ste12*Δ strains aged in fully aerated tubes with 10 ml SC containing 0.5% or 2% glucose. Lines shown in the same color are colonies from three independent transformation events; WT are *his3*Δ neutral insertions. (b) Survival curves of the WT,* ste12*Δ, and *pCUP1::GFP*‐*STE12* overexpression strains aged in SC 2% glucose in noninduced conditions (orange) or induced with 2μM (+), 5 μM (++), or 15 μM (+++) copper sulfate. Error bars are the *SEM* (*n *=* *7). (c) Cell cycle dynamics of WT and *ste12∆* populations; histograms are for cells at 0, 1, 2, and 4 hr after nitrogen starvation (left) or treatment with rapamycin (right). Cellular DNA content was measured by SYTOX‐Green staining followed by flow cytometry. Numbers indicate the percentage of cells at G1 in each population

Among the top hits of our DR regulator analysis was Ste12, a transcription factor acting on genes involved in mating or pseudohyphal growth (Dolan, Kirkman & Fields, 1989; Roberts & Fink, 1994). *STE12* is an important regulatory hub during stationary phase (Wanichthanarak, Wongtosrad & Petranovic, 2015); however, its role in stationary‐phase survival in response to nutrients has not been confirmed, as opposed to other hits such as Msn2 and Msn4 (Fabrizio et al., 2004). To further explore the functional link of Ste12 with lifespan extension, we confirmed its role in cell survival under standard conditions of full aeration (Hu, Wei, Mirisola & Longo, 2013). While the lifespan of the *ste12*Δ strain was not affected under nonrestricted 2% glucose, mutant cells showed diminished longevity in the 0.5% glucose dietary restriction medium (Figure 6a). In addition, given that our method for measuring CLS in yeast relies in the ability of stationary‐phase cells to re‐enter the cell cycle and that Ste12 is involved in cell cycle arrest, we ruled out technical artifacts using an alternative cell viability assay. Direct staining of dead and alive cells in stationary phase showed that the alive *ste12*Δ population died faster than the WT under limited glucose and, to a lesser extent, under nonrestricted conditions (Fig. S7a,b). Together, these results confirm that the lifespan effects of the *STE12* deletion are maintained regardless of the experimental conditions and methodology used to infer population survivorship in stationary phase.

High expression of a bona fide positive regulator of lifespan is expected to cause increased cell survivorship. Hence, we generated a copper‐inducible Ste12 overexpression strain with a GFP fusion to track Ste12 protein levels; the WT and *pCUP1‐STE12* strains were aged under varying concentrations of copper sulfate in 2% glucose SC medium. We found that the CLS of the *STE12*‐overexpression strain increased readily as a function of copper concentration under nonrestricted conditions (Figure 6b). Increased copper concentrations had no effect on the CLS nor growth of the WT strain (Fig. S8a,b). Importantly, GFP signal in the nucleus increased as a function of copper concentration (Fig. S9), confirming a link between lifespan and increased levels of Ste12. Together, these results unambiguously confirm that Ste12 is a novel positive transcriptional regulator of lifespan in yeast.

The TOR pathway regulates cell cycle transitions upon nutrient depletion through Rim15 activity (Pedruzzi et al., 2003). It was thus tempting to ask whether Ste12 mediates cell cycle arrest in concert with the TOR pathway. To this end, we used flow cytometry to monitor the cell cycle dynamics following treatment with the TOR‐inhibiting drug rapamycin, which is enough to trigger cell cycle arrest even in nutrient‐rich conditions. While we confirmed that the *ste12∆* mutant was impaired in cell cycle arrest upon nitrogen depletion, rapamycin treatment produced similar cell cycle profiles in *ste12*∆ and WT strains (Figure 6c). This result indicates that TOR‐mediated cell cycle arrest does not require the activity of Ste12, suggesting that this transcription factor integrates nutrient signals leading to cell cycle arrest independently of TOR signaling.

## 3 DISCUSSION

“The intrinsic nature of the aging process is essentially one of systems degradation” (Kirkwood, 2008). With a growing number of genetic aging factors in hand, the next great challenge is to understand how the mechanisms of aging and longevity are integrated to one another and to the environment. In this study, we have adapted the chronological aging paradigm in yeast to provide a quantitative and systematic description of how different dietary conditions impacts lifespan. Specifically, we have screened a collection of 3,718 gene deletion strains aged in glutamine—a preferred nitrogen source—or GABA—a nitrogen‐poor condition. Our analysis revealed that nitrogen limitation modifies the lifespan effect of 473 gene deletions (DR genes). To the best of our knowledge, this study yields the most comprehensive phenotypic compendium of genetic players involved in longevity by dietary restriction.

The classification of DR genes obtained with our kappa cluster analysis provided validation of *LE* index in the form of functional relationships. We note that we did not directly validate our list of DR genes by measuring survivorship with other CLS methodologies and calculating *LE* thereby. Functional clusters involved in longevity by nitrogen restriction included autophagy, cytosolic translation, peroxisome biogenesis, respiration, and mitochondrial function, along with novel factors such as processing of ribonucleoprotein complexes, cell‐wall organization, microtubule‐based nuclear movement, and—noteworthy—genes involved in cell cycle arrest. Furthermore, our analysis of DR genes as regulatory targets allowed the establishment of a set of ranked transcription factors (DR regulators) orchestrating the cellular response to nitrogen restriction. Strikingly, most high‐ranked DR regulators were transcription factors involved in mitotic cell cycle transitions, either by repression of Cln3 specifically in yeast daughter cells (Ace2 and Ash1) (Di Talia et al., 2009; Laabs et al., 2003), activation of ribosomal‐protein genes (Sfp1) (Marion et al., 2004), or cell differentiation in response to nutrients or pheromone (Tec1 and Ste12) (Madhani, Galitski, Lander & Fink, 1999). Top‐hit transcription factors also included, Msn2 and Msn4, two positive regulators of stress response and lifespan extension downstream of the Tor/Ras‐PKA pathways that converge on Rim15, the main protein kinase involved in cell survivorship in response to nutrients (Wei et al., 2008). We revealed that several DR regulators (Msn2, Msn4, Snf6, Tec1, and Ste12) are needed for accurate arrest of the cell cycle in response to starvation. This is in agreement with recent studies showing that dietary restriction extends the chronological lifespan of yeast, in part by regulation of the cell cycle and entry into a quiescent state (Leonov et al., 2017).

Noteworthy, deletion of *ACE2* impedes mother–daughter cell separation, thus altering the DNA content during flow cytometry, probably due to the presence of a pseudo‐filamentous growth. In addition, given that Tec1 and Ste12 are master regulators of the invasive growth pathway, it is tempting to link filamentous growth and longevity. However, the yeast background used here, S288C, bears a nonsense mutation in *FLO8* that prevents it from forming pseudohyphae, making this strain incapable of invasive/filamentous growth (Liu, Styles & Fink, 1996). Nevertheless, it remains to be addressed if Ste12 overexpression can override the deficiency of *FLO8* leading to pseudohyphae formation. While we did not confirm a causal link between cell cycle arrest defects and longevity in these knockout strains, their phenotypes suggest that cell cycle control or pseudohyphae formation is involved in lifespan extension by dietary restriction.

Importantly, the lifespan phenotypes of DR regulators were reproducible in two different modes of dietary restriction, namely nitrogen source or glucose‐concentration limitation. This was also the case for their cell cycle arrest defects: Similar gene deletion phenotypes were observed in the cell cycle dynamics triggered by both nitrogen and carbon starvation. This is relevant in the context that dietary restriction responses depend on nutrient composition (Wu, Liu & Huang, 2013) and that different sensing pathways underlie longevity by glucose or amino acid restriction (Mirisola et al., 2014). But the fact that several protocols of dietary restriction result in lifespan extension, including restriction of glucose, reduced nitrogen levels, and restriction of specific amino acids suggests that the underlying response involves overlapping mechanisms (Kennedy, Steffen & Kaeberlein, 2007). Transcription factors herein identified as DR regulators are likely part of the general machinery leading to extended lifespan regardless of the mode of dietary restriction. A next challenge is to describe how these regulators integrate signals from different nutrients and the extent to which their roles are overlapping or independent from one another.

We have provided compelling evidence that *STE12* is a positive regulator of longevity by dietary restriction. Ste12 acts downstream of two cell differentiation programs regulated by MAPK pathways, namely mating and invasive growth (Dolan et al., 1989; Roberts & Fink, 1994). Not only the *ste12*Δ strain showed diminished longevity under different experimental settings, but also *STE12* overexpression was sufficient to extend lifespan under a nonrestricted diet. Importantly, we confirmed the *ste12*Δ phenotypes in our screening settings under standard conditions in which chronological aging has been assayed elsewhere. We also found that deletion of *STE12* results in a failure to arrest the cell cycle upon nutrient starvation.

While we did not confirm a direct causal link between cell cycle arrest and extended longevity mediated by Ste12, one possibility is that regulatory elements of the mating pathway are recruited to arrest the cell cycle during dietary restriction, which in turn protects against replication stress during stationary phase, leading to increased longevity (Weinberger et al., 2007). In the presence of pheromone, the cell cycle is arrested by the action of Ste12, Far1, and the FAR complex (Far3 and Far7‐11) (Kemp & Sprague, 2003); Far1 and Far3 are direct targets of Ste12 (Lefrançois et al., 2009). Concomitantly, deletion of *FAR7*,* FAR8*, and *FAR11* in our primary screen resulted in diminished lifespan extension. An alternative possibility is that Ste12 mediates cell cycle arrest and longevity through its association with Tec1. This transcription factor promotes cell cycle progression through induction of *CLN1* (Madhani et al., 1999). Hence, association with Ste12 by filamentous growth pathway nutrient signaling (Madhani et al., 1999) could prevent Tec1‐mediated progression of the cell cycle, resulting in increased longevity.

The TOR pathway regulates cell cycle transitions upon nutrient depletion through activity of the Rim15 kinase (Pedruzzi et al., 2003). However, regulation of the cell cycle shows poor correlation with the short‐lived phenotype of Rim15‐deficient cells; other TOR/Rim15‐dependent mechanisms such as carbohydrate storage seem to play a more important role (Cao et al., 2016). In agreement, we found that Ste12 regulation of cell cycle is not under the control of the TOR pathway, as rapamycin treatment did not affect G1 arrest in a *ste12*∆ background, suggesting that Ste12 controls cell cycle transitions in response to nutrients in a TOR‐independent manner. Hence, it is possible that Ste12 regulates lifespan through other mechanisms. Noteworthy, *STE11*, an upstream component of the pheromone pathway, is also implicated in cell cycle and lifespan regulation (Cao et al., 2016). This supports the idea that Ste12 controls cell cycle transitions in response to nutrients and that the emergence of a G1‐arrested population is necessary for lifespan extension.

Whether the longevity roles of the DR regulators are conserved is still an open question. For instance, *STE12* has no clear homolog in animals; however, transcriptional networks can be rewired through evolution, leading to changes in the regulation exerted by specific regulators, while the downstream targets remain associated (Sorrells, Booth, Tuch & Johnson, 2015). In addition, the yeast three‐kinase module regulating MAPK pheromone and invasive growth pathways are conserved in other organisms (Widmann, Gibson, Jarpe & Johnson, 1999). In particular, *KSS1* and *FUS3* are key members of the MAPK pathway that regulates cell differentiation programs in yeast (Bardwell, 2004), while their mammalian counterpart *MAPK1* is central to the development of several age‐associated diseases in mammals (Carlson, Silva & Conboy, 2008). Thus, the study of targets downstream the MAPK pathway in yeast might bring important insights into the regulation of aging in other eukaryotes, including humans.

Our study provides a much‐needed comprehensive picture of the mechanisms of lifespan extension by dietary restriction, underscoring key links between nutrient sensing, the cell cycle arrest machinery, and longevity in yeast. Our approach can be readily applied to other genetic, environmental, or pharmacological perturbations, providing a systematic framework to describe aging networks in a simple tractable system. Other cross‐talks among downstream cellular processes and their transcriptional regulators may remain to be uncovered, which will shed further light to the genetic wiring of aging cells.

## 4 EXPERIMENTAL PROCEDURES

### 4.1 Strains and media

Fluorescent single‐gene deletion strains are prototrophic haploids (*MAT*a *xxx*Δ*::kanMX4 PDC1‐mcherry‐CaURA3MX4 can1*Δ*:STE2pr‐SpHIS5 lyp1*Δ *his3*Δ*1 ura3*Δ*0 LEU2*) derived from crossing the *MAT*α YEG01‐RFP SGA‐starter with 4,340 viable deletion strains from the *MAT*a BY4741 collection from the Saccharomyces Genome Deletion Project (Garay et al., 2014). All de novo single‐gene deletions were generated in the YEG01‐RFP parental strain (*MAT*α *PDC1‐mcherry‐CaURA3MX4 can1*Δ*:STE2pr‐SpHIS5 lyp1*Δ *his3*Δ*1 ura3*Δ*0 LEU2*) by direct gene replacement with the *natMX4* module conferring resistance to clonNAT. The Ste12 overexpression strain was generated by inserting the *CUP1* promoter and GFP fusion construct from plasmid pYM‐N4 in the 5′ region of *STE12* ORF (Janke et al., 2004).

Nonrestricted (NR) aging medium contained 0.17% yeast nitrogen base (YNB) without amino acids and ammonium sulfate, 2% glucose, 0.07% amino acid supplement mix (Data S4), and 25 mM glutamine as nitrogen source (see Table S1 for media summary). Dietary‐restricted (DR) aging medium was prepared substituting glutamine with 25 mM of GABA. All other nitrogen sources tested (ammonium sulfate, methionine, asparagine, phenylalanine, leucine, isoleucine, and valine) were also supplemented at 25 mM, as this results in an equivalent culture yield as the commonly used 0.5% ammonium sulfate. The choice of a nonpreferred nitrogen source for DR instead of limited glucose concentrations overcomes the dramatic metabolic changes due to glucose repression in yeast (Kresnowati et al., 2006), while facilitating the parallel characterization of stationary‐phase cultures in low volumes, given that cell yields are similar under nonrestricted and dietary restriction conditions. SC medium used for glucose restriction was 0.17% yeast nitrogen base (YNB) without amino acids, 0.2% amino acid supplement mix, and 0.5% or 2% glucose. Outgrowth cultures for all CLS experiments were performed in low fluorescence medium (Garay et al., 2014). No media used here contain any form of buffering.

Nitrogen starvation medium for cell cycle progression experiments was 2% glucose and 0.17% yeast nitrogen base without amino acids and ammonium sulfate. Glucose starvation medium contained no glucose and 0.67% yeast nitrogen base without amino acids and 0.2% amino acid supplement mix. All media recipes and preparation protocols are provided in detail in S4.

### 4.2 Automated competition‐based CLS screens and data analysis

Fresh cultures of 4,340 tagged gene deletion strains were replicated in 96‐well plates (Corning 3585) with 150 μl of NR or DR aging medium. Deletion strains with slow growth and/or low fluorescence signal were discarded, only 4,050 strains passed this filter. In addition, several strains were lost during the subsequent steps of the automated experimental setup, resulting in the recovery of only 3,718 deletion strains that were tested under both glutamine and GABA. Saturated cultures were mixed with a CFP‐labeled WT reference strain in a 2RFP:1CFP ratio for all mutants and WT controls. The latter was performed to increase the dynamic range of mutant measurements, as many of them displayed lower fluorescence than the WT. Importantly, we did not use a dye‐swap strategy limiting our capacity to establish links between mutant cells yield and fluorescence intensity. Mixed cultures were then replicated by pinning into 96 deep‐well plates (Nunc 260251), containing 700 μl of NR or DR aging medium, and grown at 30°C and 70% relative humidity, without shaking, in an automated system (Tecan Freedom EVO200) integrated to a multilabel plate reader (Tecan M1000). Four days after inoculation into deep‐well plates, cultures were fully re‐suspended and 5 μl outgrowth cultures were inoculated every other day into 150 μl of fresh low fluorescence medium (Data S4) with the aid of an automated robotic arm. Absorbance at 600 nm (OD_600_) and fluorescence (*RFP* and *CFP*) measurements were taken every 150 min throughout 14 hr with a Tecan multiplate reader. An apparent survival coefficient, *s*, and its standard error, σ_*s*_, were obtained from the slope of the linear regression (Robustfit, Matlab) of the log ratio of *RFP* to *CFP* signal at a fixed interpolation time point in the outgrowth culture (10 hr), throughout 21 days in stationary phase. We observed no significant differences between the distribution of standard errors among the different phenotypic categories (Fig. S2). All raw data collected in both genomewide screens are available at http://www.langebio.cinvestav.mx/deluna/Campos2018/.

We have shown that the choice of reference strain does not impact CLS effects of the mutants (Garay et al., 2014). Other experimental schemes for culture mixing could be tested, such as mixing reference and mutant strains after separate aging to avoid any kind of strain‐strain interaction; hence, care must be taken when interpreting individual cases. While CLS effects of mutants aged under low aeration show no drastic changes compared to highly aerated cultures (Garay et al., 2014), we note that WT CLS decreases substantially in aerated conditions, and thus, mutant‐specific effects might exist, specially, those involved in aerobic or anaerobic metabolism.

### 4.3 Scoring CLS phenotypes and lifespan extension coefficients

Short‐ and long‐lived knockouts under NR or DR were determined by assigning a *Z*‐score ($Z=\frac{s_{\mathrm{mut}-\mu_{\mathrm{wt}}}}{\sigma_{\mathrm{wt}}}$ to each mutant's *s* coefficient; the distribution's mean (μ) and standard deviation (σ) of the population were taken from the measurement of 264 WT_RFP_/WT_CFP_ independent co‐cultures under either condition, assuming equal distribution of errors in the mutants and the WT. Two‐tailed *p*‐values were obtained from each *Z*‐score to compute a false‐discovery rate (FDR); we assigned significant phenotypes using a 5% FDR. While the mutants were not replicated under the same condition, the correlation of technical replicates has been measured previously (*r *=* *.88, Pearson) (Garay et al., 2014).

The effect of dietary restriction on the gene deletion strains relative to the WT was evaluated by calculating their relative lifespan extension defined as $LE=\frac{s_{\mathrm{DR}}+1}{s_{\mathrm{NR}}+1}$, where *s*_NR_ and *s*_DR_ are the *s* coefficients of a given deletion strain obtained from the screen under NR and DR, respectively. A *Z*‐score was assigned to the *LE* of each mutant compared to the distribution of *LE* values of 264 independent WT reference experiments; significant *LE *< 1 and *LE *> 1 values were assigned at a 5% FDR.

### 4.4 Small‐scale CLS assay measured by outgrowth kinetics

Selected strains were grown individually in the indicated nonrestricted or dietary restriction media for 48 hr at 30°C 200 rpm in aerated tubes, then transferred to 96‐well plates. In this step, each strain tested was inoculated several times in parallel from a single culture stock, and the same setup was used throughout all our single culture assays performed in deep‐well plates. These plates were replicated onto 96 deep‐well plates containing 700 μl of NR or DR medium and left for the entire experiment at 30°C and 70% relative humidity without shaking. From here on, all experimental steps were performed in an automated robotic station (Tecan Freedom EVO200). After 4 days, 10 μl aliquots were taken with an automated 96‐channel pipetting arm to inoculate 96‐well plates containing 150 μl of low fluorescence medium. OD_600_ was obtained in a plate reader (Tecan M1000) every 1.5 hr until saturation was reached; this first outgrowth‐kinetics curve was regarded as the first time point (*T*_0_, age = 0 days). Sampling was repeated every 2–3 days for 24–28 days. From this outgrowth kinetics, we extracted the doubling time and the time shift to reach midexponential phase (OD_600_ = 0.3) that occurred between the first day of measurements (*T*_0_) and each day in stationary phase (*T*_*n*_). Relative cell viability was calculated from these data, as reported by Murakami et al. (2008).

Viability data points relative to *T*_0_ were used to plot a survival curve, which was fitted (all data points) to an exponential decay model ($N\left(T\right)=N_{0}e^{-rT}$) where *N*_0_ is the percentage of viability at *T*_0_, *T* is time in days, and *r* is the rate of death. For validation of CLS effects, mutants were taken from the RFP‐tagged deletion collection (or generated de novo, when indicated) and viability was assayed to calculate death rates in at least seven experimental replicates which were compared to replicates of a WT strain; significant CLS effects were considered using a *p *<* *.05 cutoff (*T* test).

### 4.5 CLS assay in standard aeration conditions

Standard culture conditions were used, as described (Hu et al., 2013). In brief, pre‐inoculums from three different colonies of each strain were set in 5‐ml SC medium for 24 hr, and these were diluted 1:100 v/v in 10 ml SC with 2% glucose or 0.5% glucose aging medium in 50‐ml tubes for 48 hr with shaking (200 rpm) at 30°C. Viability at each measuring point was obtained by monitoring the change in outgrowth‐kinetics parameters with time in stationary phase using three technical replicates for each colony tested, as described above.

### 4.6 Visualization of functional clusters

Gene ontology (GO) associations and phenotype terms were downloaded from the *Saccharomyces* Genome Database (SGD, last updated December 2016) to build two *m* by *n* matrixes, where *m* is the number of DR genes (219 and 253 for *LE *< 1 and *LE *< 1, respectively) and *n* is the number of GO and phenotypic terms (1,748). Each term was used to evaluate the overall agreement between gene pairs to calculate Cohen's *kappa* ($\kappa =\frac{\text{Pra}(a)-\mathrm{Pr}(e)}{1-\text{Pr}(e)}$). Where *Pra*(*a*) is the number of associated terms and not associated terms that each gene pair shares, divided by the total number of terms downloaded from SGD in the matrix (denominator is the same for any gene pair), and *Pr*(*e*) is the hypothetical probability for each member of the gene pair to be associated by chance.

A matrix representing the agreement between each gene pair was built with the *kappa* values. Gene pairs that showed κ* *> 0.35 were regarded as likely similar, according to previous reported thresholds for large datasets (Huang, Lempicki & Sherman, 2009). Gene pairs formed in the first step were used as cluster seeds to form larger groups of genes; that is, groups of genes that shared at least 50% of their members merged in subsequent iterative steps, thus creating larger groups in each iteration until only groups with dissimilar members remained. Clusters with at least four elements were manually named by inspection in the SGD and GO enrichment. All procedures related to Kappa calculation and analysis were performed with Matlab; input genes gene association data and Matlab scripts are available at http://www.langebio.cinvestav.mx/deluna/Campos2018/. Network representation was created using Cytoscape; edges between nodes represent kappa agreement above the established threshold (κ > 0.35).

### 4.7 TFRank analysis

TFRank was used at http://www.yeastract.com/formrankbytf.php using the *TF Rank* algorithm option (Gonçalves et al., 2011), with a heat diffusion coefficient of 0.25. All DR genes were introduced as Target ORF/Genes, with all transcription factors selected (*n *=* *197). The output file includes all transcription factors reported, ranked, and weighted for regulation of the input DR genes list (Data S5).

### 4.8 Flow cytometry analysis of stationary‐phase fluorescence signal dynamics

Twenty deletion strains and four WT replicates were aged in deep‐well plates containing with SC medium with GABA nitrogen source. All cultures were inoculated at different times to achieve ages from three to nineteen days in the same plate. Cell cytometry (LSRFortessa™, Becton Dickinson) was performed after inoculating 5 μl of the original cultures into 150 μl of YNB‐low fluorescence medium; this outgrowth was incubated for 11 hr at 30°C. With the aid of a high‐throughput sampler, 20,000 events were collected for each culture in the 96‐well plate (20 mutants and four WT replicates, eight time points each); mCherry was excited with a 561‐nm laser, and fluorescence was collected through a 586/15 band‐pass filter.

### 4.9 Alive/dead staining assay

We used the same scheme of 96 deep‐well plates (one plate per replicate) to age cells in SC with 0.5% or 2% glucose. Each day, a single well of each strain was collected. Cells were centrifuged, washed, and dyed with LIVE/DEAD^®^ FungaLigth™ Yeast Viability Kit, following manufacturer's instructions. Propidium iodide (IP) and Syto^®^9 fluorescence were measured by cell cytometry (LSRFortessa™, Becton Dickinson) at early stationary phase (4 days after inoculation) and at different time‐points until 21 days in stationary phase. IP was excited with a 591‐nm laser, fluorescence was collected through a 586/15 band‐pass filter; Syto9 was excited with a 488‐nm laser, and fluorescence was collected through 525/50 band‐pass and 505LP emission filters. Cell viability percentage was obtained by staining dead and alive cells with SYTO9 and subtracting the number of dead cell events only as stained by propidium iodide.

### 4.10 Cell cycle assays

WT and mutant strains were grown in flasks containing 50 ml of NR aging medium at 30°C and shaken at 200 rpm until midlogarithmic phase (OD_600_ ≅ 0.5). Cells were centrifuged, washed twice with sterilized water, and transferred to nitrogen or glucose starvation medium. For a rapamycin‐induced arrest, 10 nM rapamycin was directly added to mid‐log phase cell cultures. Samples were taken at the moment of either transfer to starvation medium or rapamycin addition (time 0) and 1, 2, and 4 hr after that time point. Fixation and dying with SYTOX™Green were performed as described elsewhere. Cells were analyzed by flow cytometry (LSRFortessa™, Becton Dickinson); SYTOX‐Green was excited with a 488‐nm laser, and fluorescence was collected through a 525/50 band‐pass filter.

## CONFLICT OF INTEREST

The authors declare that they have no competing interests.
