# Supplementary material for: Genomewide mechanisms of chronological longevity by dietary restriction in budding yeast
Source: Aging Cell. 2018 Mar 25;17(3):e12749. doi: 10.1111/acel.12749 (PMC5946063; doi:10.1111/acel.12749)
Supplement: Supplementary file 1 [file ACEL-17-e12749-s001.pdf]

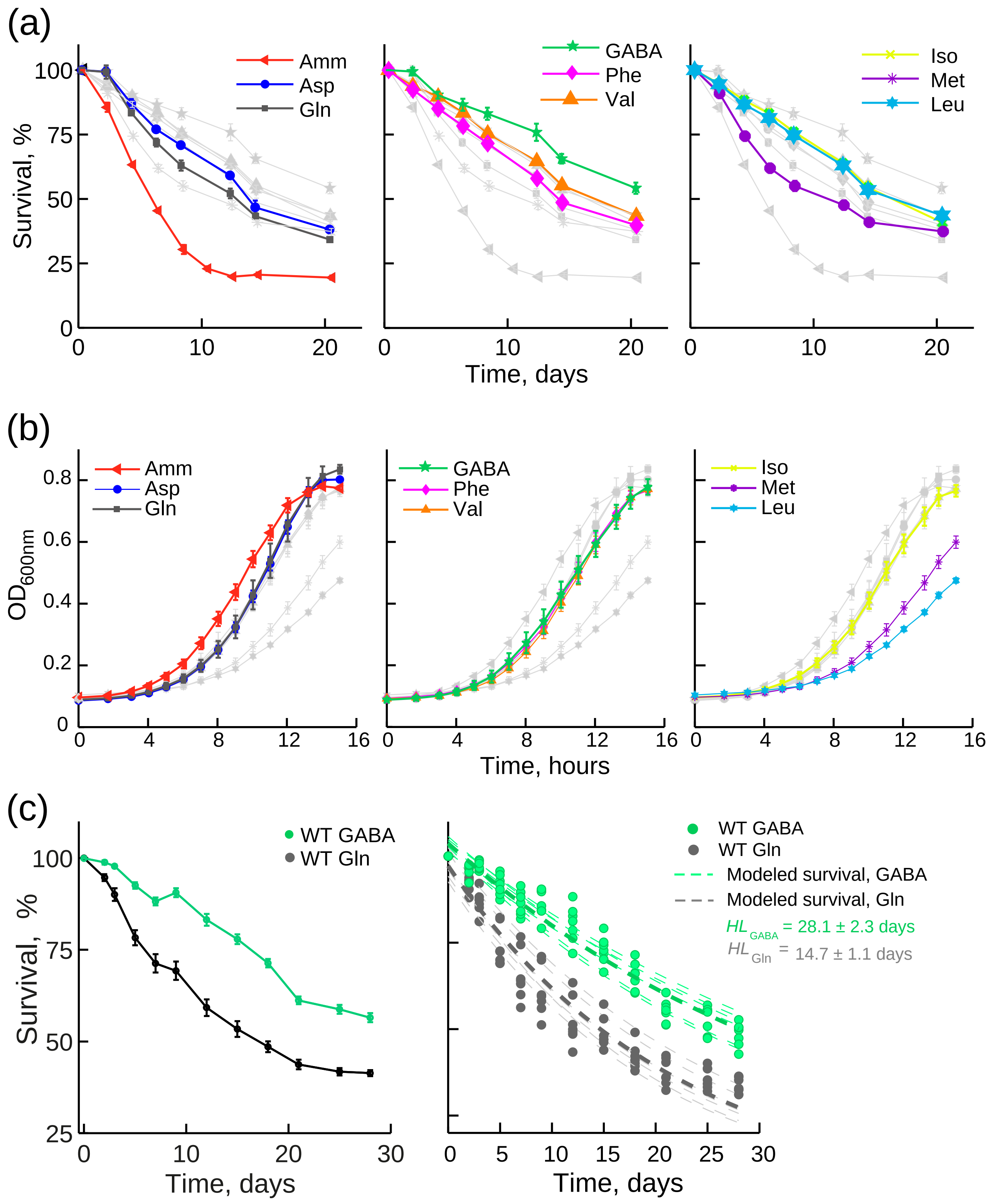

Figure S1. The CLS of yeast is affected by the quality of the nitrogen source in the aging medium. (**a**) Survival curves of the WT strain obtained with a CLS method based on outgrowth kinetics (adapted from Murakami *et al.* 2008); survival curves on different nitrogen sources are shown according to transcriptomic response to supplementation with these amino acids (Godard *et al*. 2007): rich (left), intermediate (middle) and poor nitrogen sources (right). All of these conditions were tested in a single 96-deepwell plate, to avoid experimental batch variation, at least 6 replicates were aged and measured for each condition. (**b**) Growth kinetics of the WT strain under the different nitrogen sources. (**c**) An independent experiment for survival of the WT strain aged under Glutamine and GABA (left); error bars are the S.E.M. (*n*=6). Data of each survival curve was adjusted to an exponential decay model to obtain the decay rate and half life under each condition (*HL*, right).
