# Supplementary material for: Genomewide mechanisms of chronological longevity by dietary restriction in budding yeast
Source: Aging Cell. 2018 Mar 25;17(3):e12749. doi: 10.1111/acel.12749 (PMC5946063; doi:10.1111/acel.12749)
Supplement: Supplementary file 2 [file ACEL-17-e12749-s002.pdf]

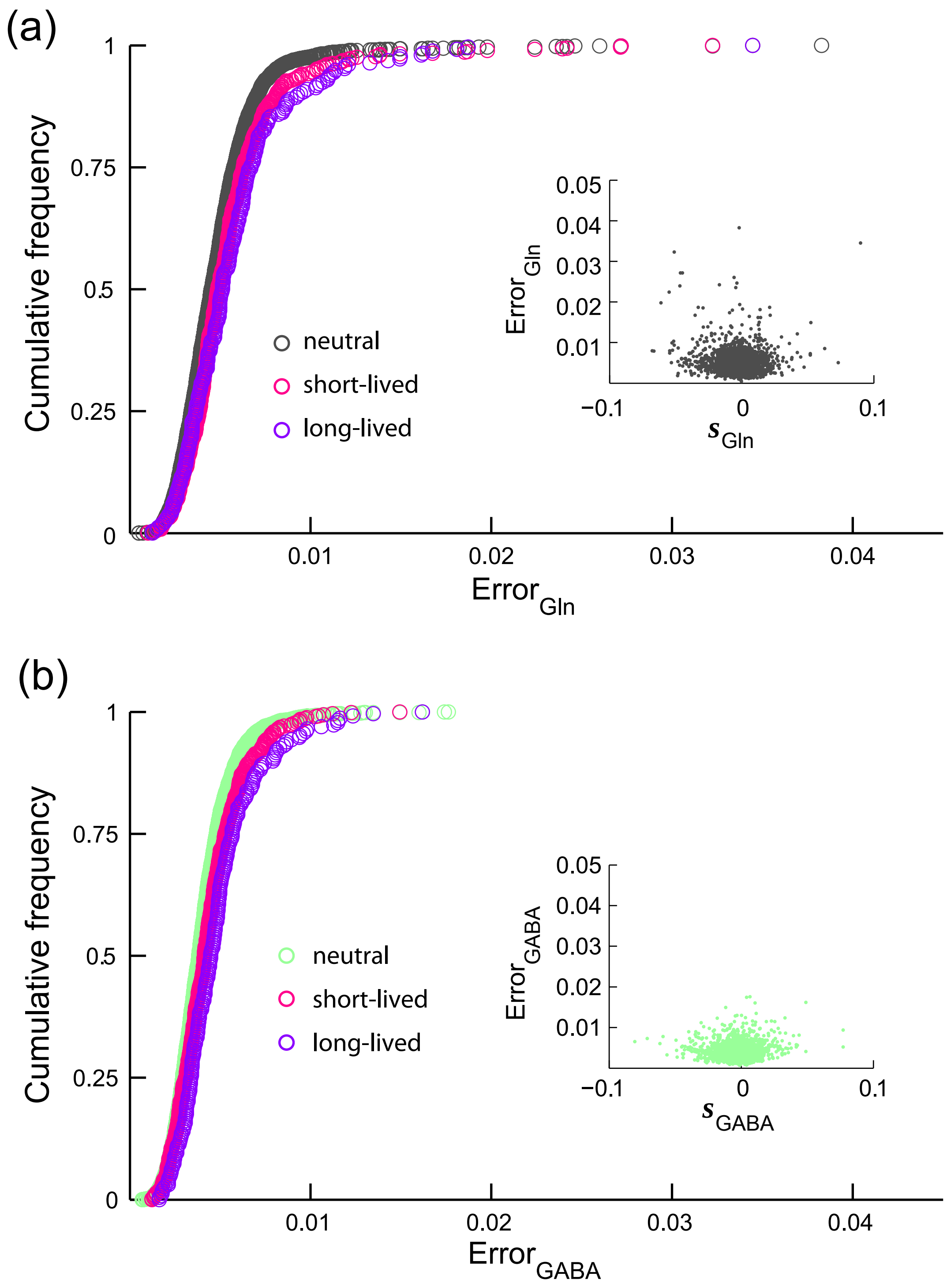

Figure S2. The error in the fitted model shows no correlation with lifespan phenotypes scored nor estimated survival coefficients. Cumulative frequencies of the errors in the survival coefficients measured under **(a)** glutamine or **(b)** GABA for neutral, short-lived, and long-lived knockout strains. The error is from the coefficients of the linear regression equation calculated by robust fit (Matlab). Insets show the error in the fit as a function of the survival coefficient, *s*.
