# Supplementary material for: Genomewide mechanisms of chronological longevity by dietary restriction in budding yeast
Source: Aging Cell. 2018 Mar 25;17(3):e12749. doi: 10.1111/acel.12749 (PMC5946063; doi:10.1111/acel.12749)
Supplement: Supplementary file 3 [file ACEL-17-e12749-s003.pdf]

Fig S3

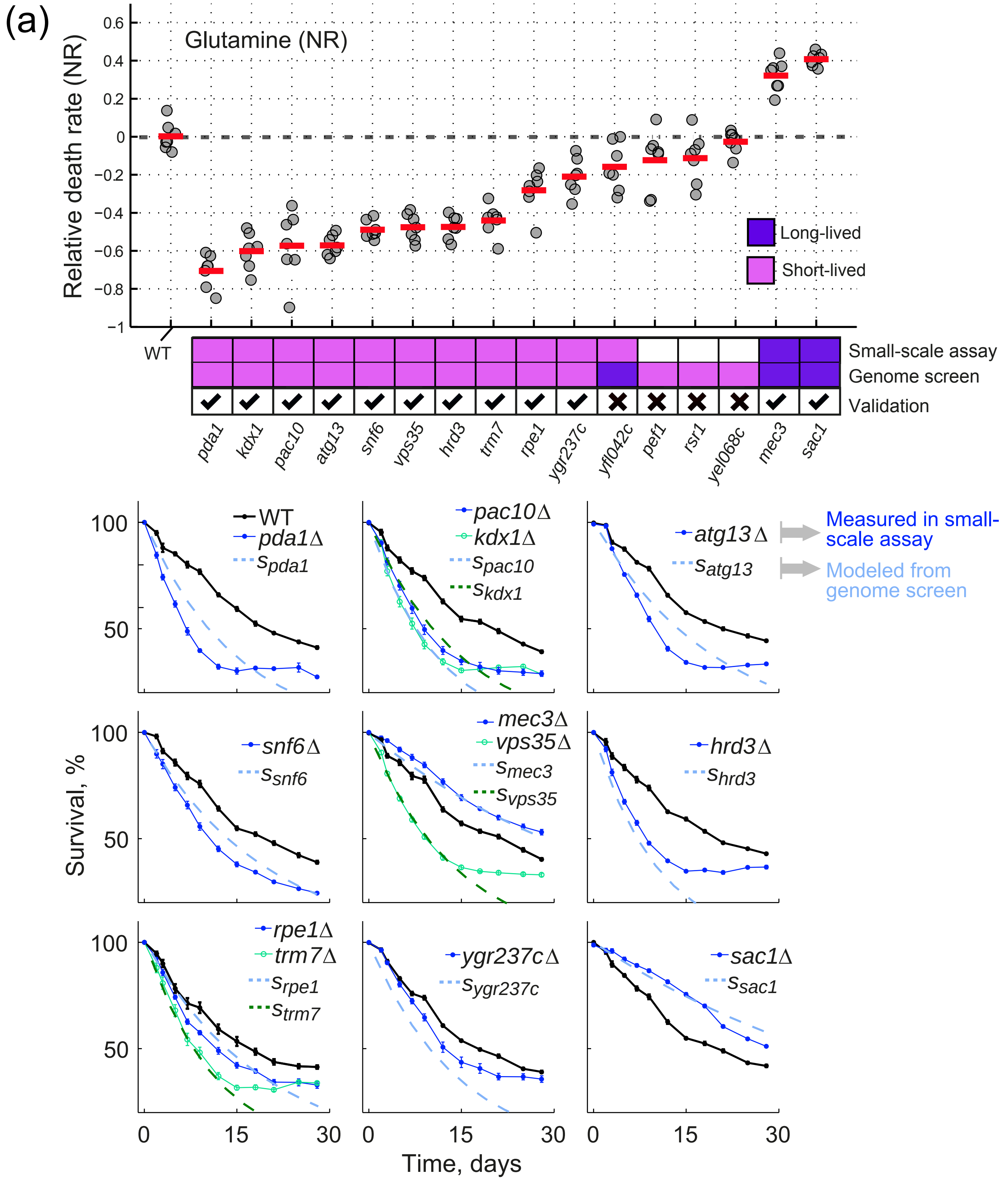

Figure S3. Validation of CLS hits from the genome-wide screen. (**a**) Top plot shows the relative rates of death of WT and mutant strains, -1**ln*(r_x / r_{wt}), aged under glutamine medium, measured by outgrowth kinetics (Murakami *et al.* 2008). Death rates were obtained by fitting the survival curves to an exponential decay model, as shown in Figure S1C. Hits from the genome-wide screen were considered valid when the death rate of the corresponding mutant was consistent (short-lived or long-lived) and statistically different from that of the WT (p<0.05, *T*-test). Bottom plots show the survival of single cultures (solid lines); only validated strains are shown, along with the specific WT of each experimental batch. Error bars are the S.E.M. (n=7). In addition, survival coefficients (s) from the genome-wide screen where used to model a survival curve for the corresponding knockout strain (dashed lines). (*Continued in next page*)

Fig S3

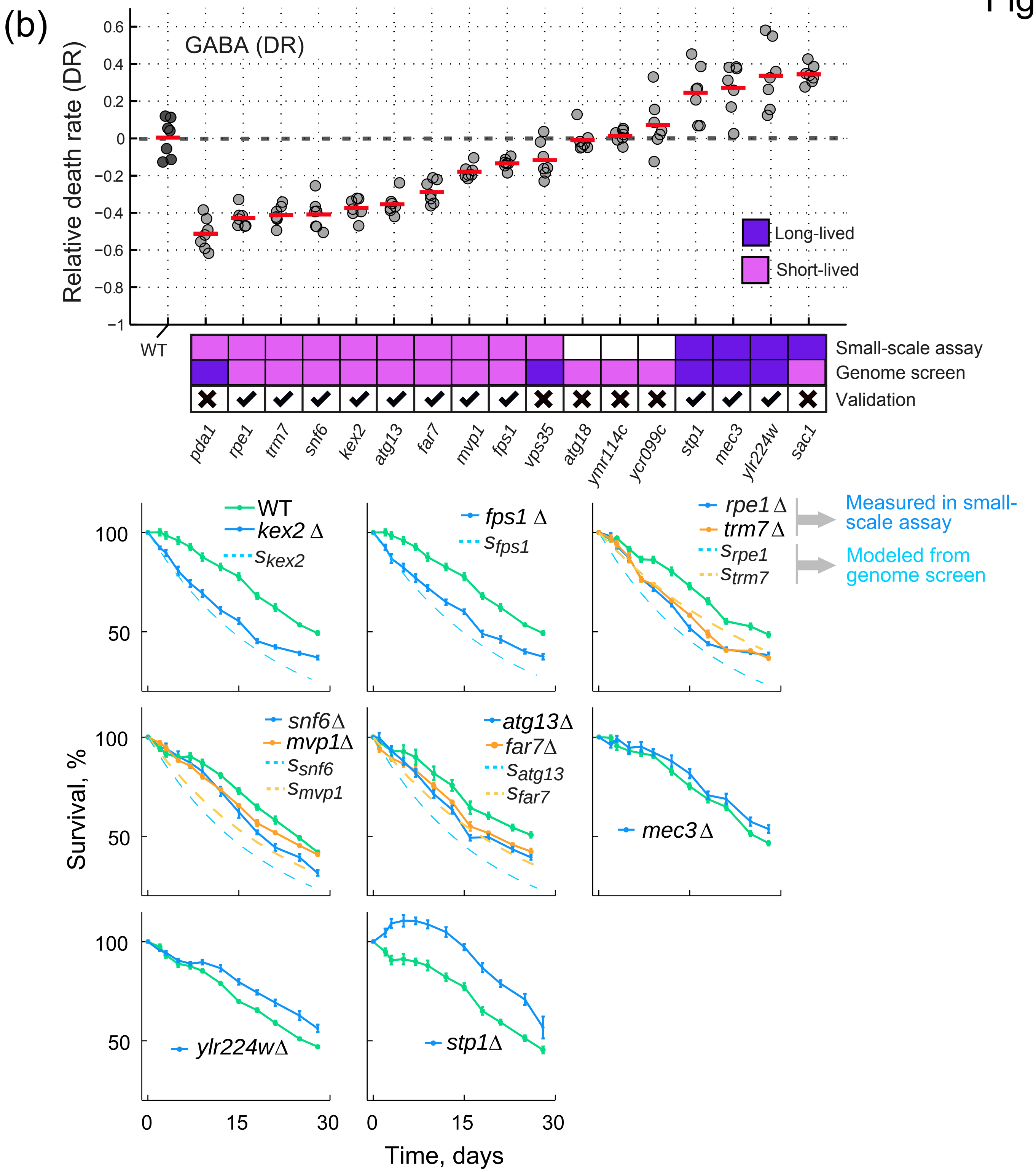

Figure S3. Validation of CLS hits from the genome-wide screen. (**b**) Top plot shows the relative rates of death of WT and mutant strains, -1**ln*(r_x / r_{wt}), aged under GABA medium, measured by outgrowth kinetics (Murakami *et al.* 2008). Death rates were obtained by fitting the survival curves to an exponential decay model, as shown in Figure S1C. Hits from the genome-wide screen were considered valid when the death rate of the corresponding mutant was consistent (short-lived or long-lived) and statistically different from that of the WT (p<0.05, *T*-test). Bottom plots show the survival of single cultures (solid lines); only validated strains are shown, along with the specific WT of each experimental batch. Error bars are the S.E.M. (n=7). In addition, survival coefficients (s) from the genome-wide screen where used to model a survival curve for the corresponding knockout strain (dashed lines). Given the slow death rate of yeast cells in GABA, positive survival coefficients s>0.02 cannot be used to model survival curves, and are therefore not show for the long-lived *mec3*Δ, *ylr224w*Δ, and *stp1*Δ knockouts in GABA. (*Continued from previous page*)
