# Supplementary material for: Genomewide mechanisms of chronological longevity by dietary restriction in budding yeast
Source: Aging Cell. 2018 Mar 25;17(3):e12749. doi: 10.1111/acel.12749 (PMC5946063; doi:10.1111/acel.12749)
Supplement: Supplementary file 4 [file ACEL-17-e12749-s004.pdf]

Fig S4

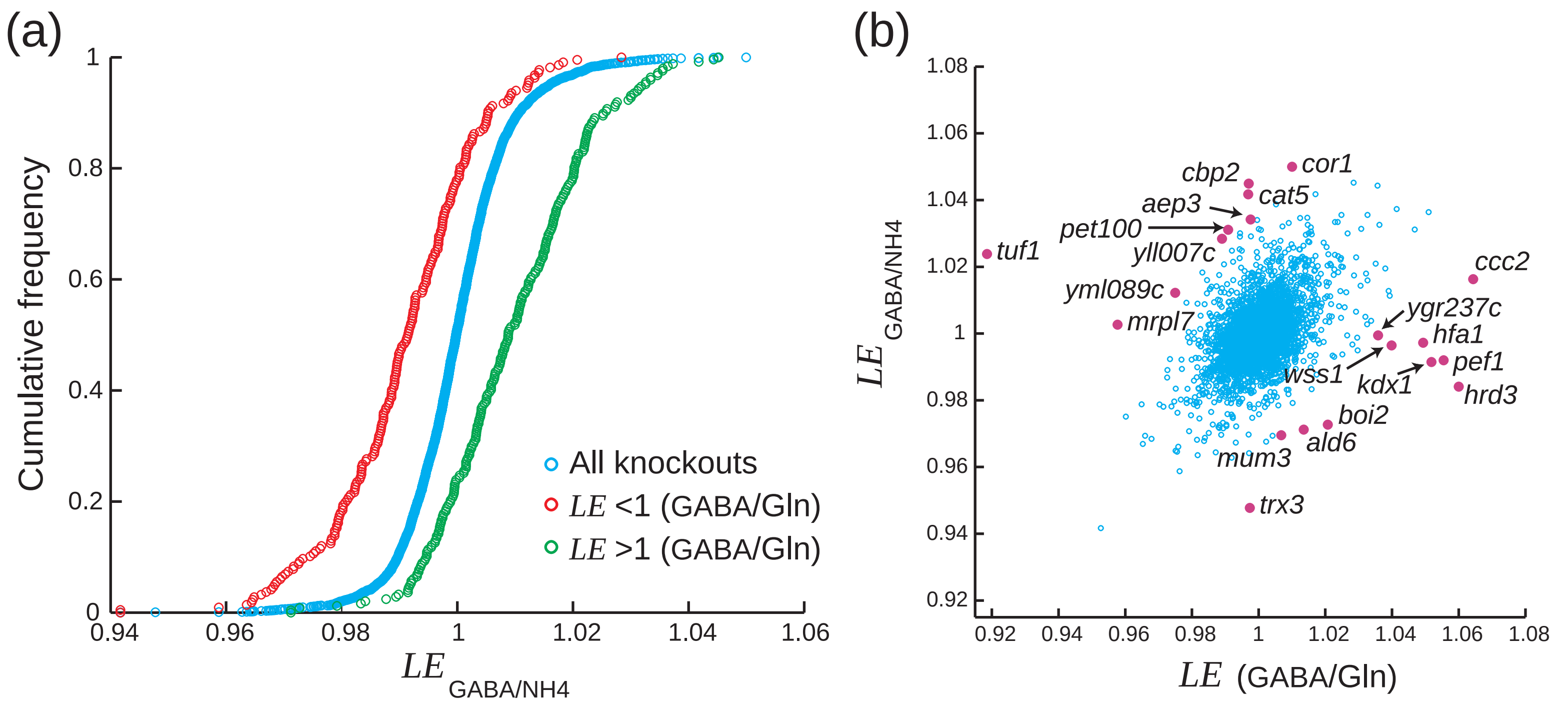

(c)

Gene	GO: Cellular component	Gene	GO: Cellular component
$LE_{NH4}>LE_{Gln}$		$LE_{NH4}<LE_{Gln}$	
TUF1	Mitochondria	*HRD3*	Endoplpasmic reticulum
CBP2	Mitochondria	*PEF1*	Cytoplasm, Bud neck
MRPL7	Mitochondrial large ribos. subunit	*KDX1*	Unknown
CAT5	Mitochondrial inner membrane	*HFA1*	Mitochondria
PET100	Mitochondrial inner membrane	*TRX3*	Mitochondria
YLL007C	Mitochondria, Plasma membrane	*BOI2*	Bud neck
COR1	Mitochondrial resp. chain complex III	*ALD6*	Mitochondria
YML089C	Unknown	*CCC2*	Trans-Golgi vesicle membrane
AEP3	Mitochondrial inner membrane	*WSS1*	Vacuole, nuclear envelope
		MUM3	Unknown
		YGR237C	Cytoplasm

Figure S4. $LE_{GABA/NH4}$ values were obtained for 3,681 knockout strains (99% of our data set) by comparing the survival coefficients measured under GABA (dietary restriction, this study) and an alternative non-restricted condition using ammonium sulfate as the sole nitrogen source (data from Garay *et al*. 2014). It must be noted that, while data in ammonium was obtained in mutants aged in buffered medium, the SC media used in this study (GABA and glutamine nitrogen sources) were not buffered. (**a**) Cumulative distributions of $LE_{GABA/NH4}$ values for all knockout strains (blue), strains with diminished (LE<1) and enhanced (LE>1, green) lifespan extension defined form the GABA/glutamine comparison. (**b**) Direct comparison of the LE values obtained with the two conditions of non-restricted nitrogen. The top 20 genes with highest LE differences are highlighted (magenta). (**c**) Sub-cellular localization of the set of highly condition-specific genes in panel (b).
