# Supplementary material for: Genomewide mechanisms of chronological longevity by dietary restriction in budding yeast
Source: Aging Cell. 2018 Mar 25;17(3):e12749. doi: 10.1111/acel.12749 (PMC5946063; doi:10.1111/acel.12749)
Supplement: Supplementary file 5 [file ACEL-17-e12749-s005.pdf]

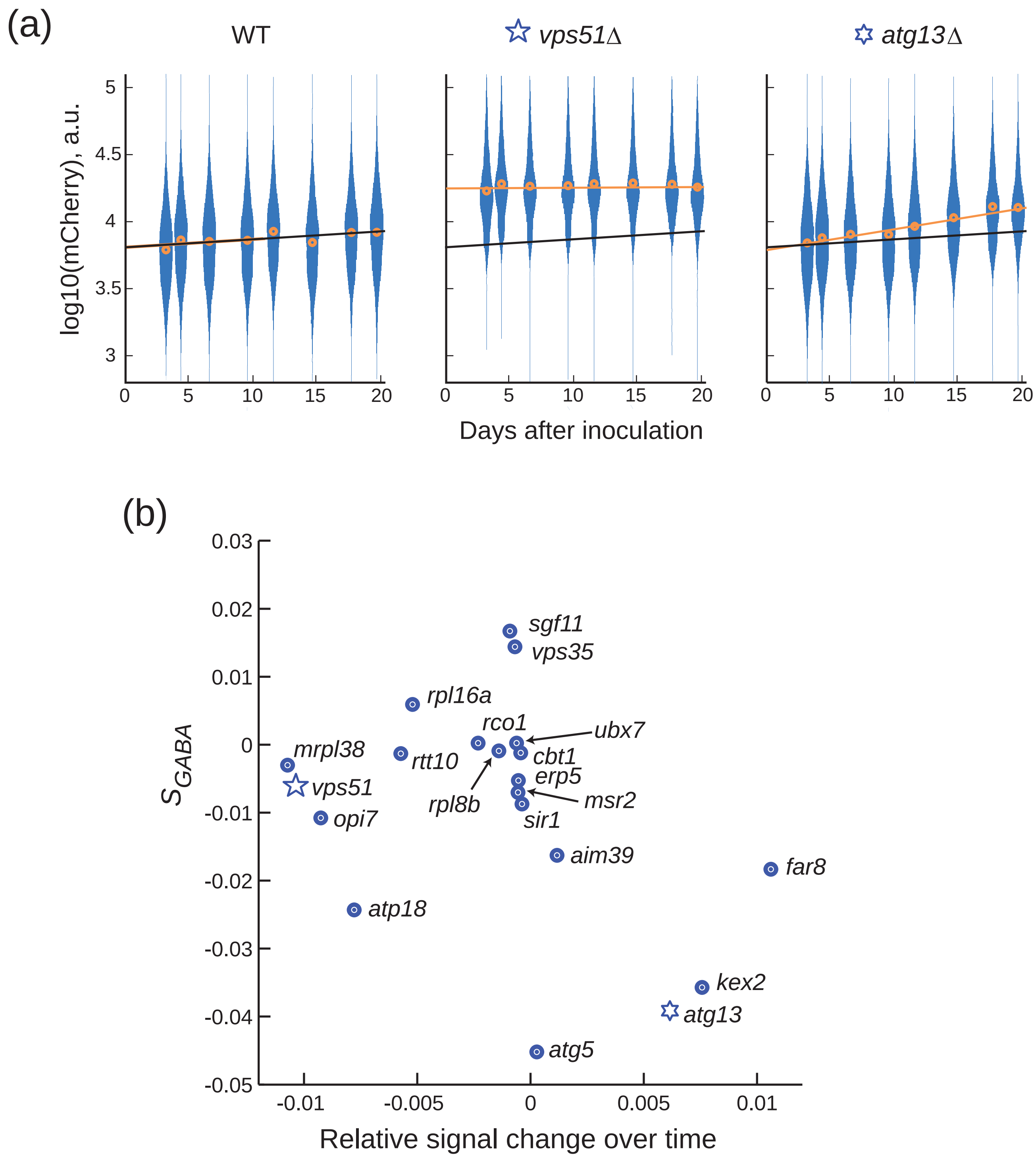

Figure S5. Changes in fluorescent-protein signal as a function of time in stationary phase. (**a**) Violin plots for the entire distributions of mCherry signal in 20,000 events (cells) measured by flow cytometry. The average signal of each population (orange circles) over time in stationary phase was adjusted to a linear fit in the WT (black line) and in each mutant strain (orange line); only two extreme cases, *vps51*Δ and *atg13*Δ, are shown. (**b**) For all 20 deletion strains tested, the rate of fluorescence change relative to the WT is plotted against the survival coefficient of the corresponding mutant strain (*r*=-0.39, *p*=0.092, Pearson correlation).
