# Supplementary material for: Genomewide mechanisms of chronological longevity by dietary restriction in budding yeast
Source: Aging Cell. 2018 Mar 25;17(3):e12749. doi: 10.1111/acel.12749 (PMC5946063; doi:10.1111/acel.12749)
Supplement: Supplementary file 6 [file ACEL-17-e12749-s006.pdf]

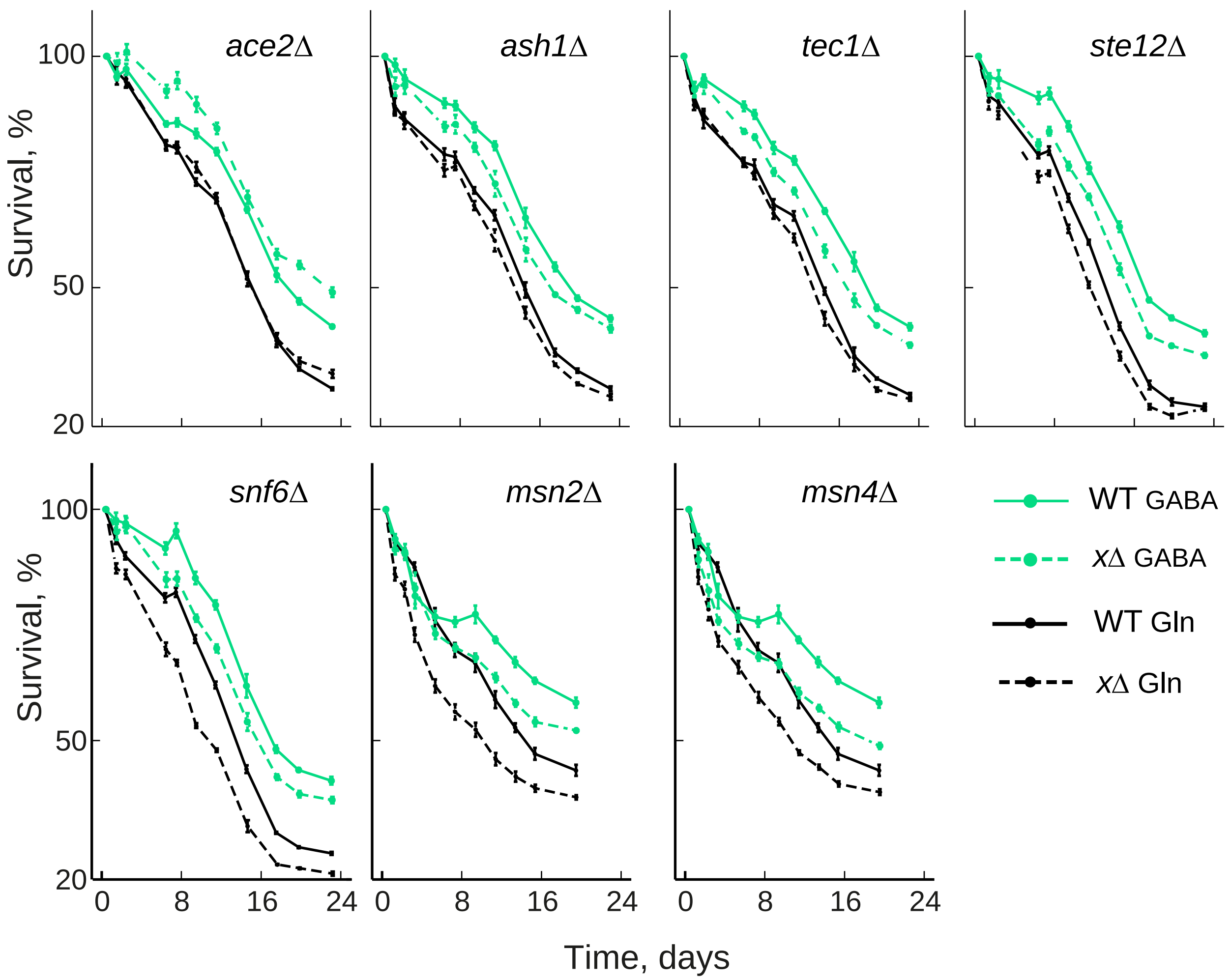

Figure S6. CLS of gene deletions of transcription factors that regulate dietary-restriction genes under different nitrogen sources. Survival curves of WT and gene-deletion strains aged in SC medium with 2% glucose and glutamine (non-restricted, black lines) or GABA nitrogen source (dietary restriction, green lines). Deletion strains (discontinuous lines) are for genes coding for transcription factors Ace2, Ash1, Tec1, Ste12, Snf6, Msn2, and Msn4. These strains are the exact same strains shown in Fig4B; the experimental batch shown here did not include the *bas1*Δ strain. The msn2Δ and msn4Δ strains were characterized in a separate batch along with the WT shown in these two panels. At least seven replicates were aged in parallel from a single stock for each deletion strain (a single colony verified from one transformation event) in the same deep-well plate. Error bars are the S.E.M. (n=7).
