# Supplementary material for: Genomewide mechanisms of chronological longevity by dietary restriction in budding yeast
Source: Aging Cell. 2018 Mar 25;17(3):e12749. doi: 10.1111/acel.12749 (PMC5946063; doi:10.1111/acel.12749)
Supplement: Supplementary file 7 [file ACEL-17-e12749-s007.pdf]

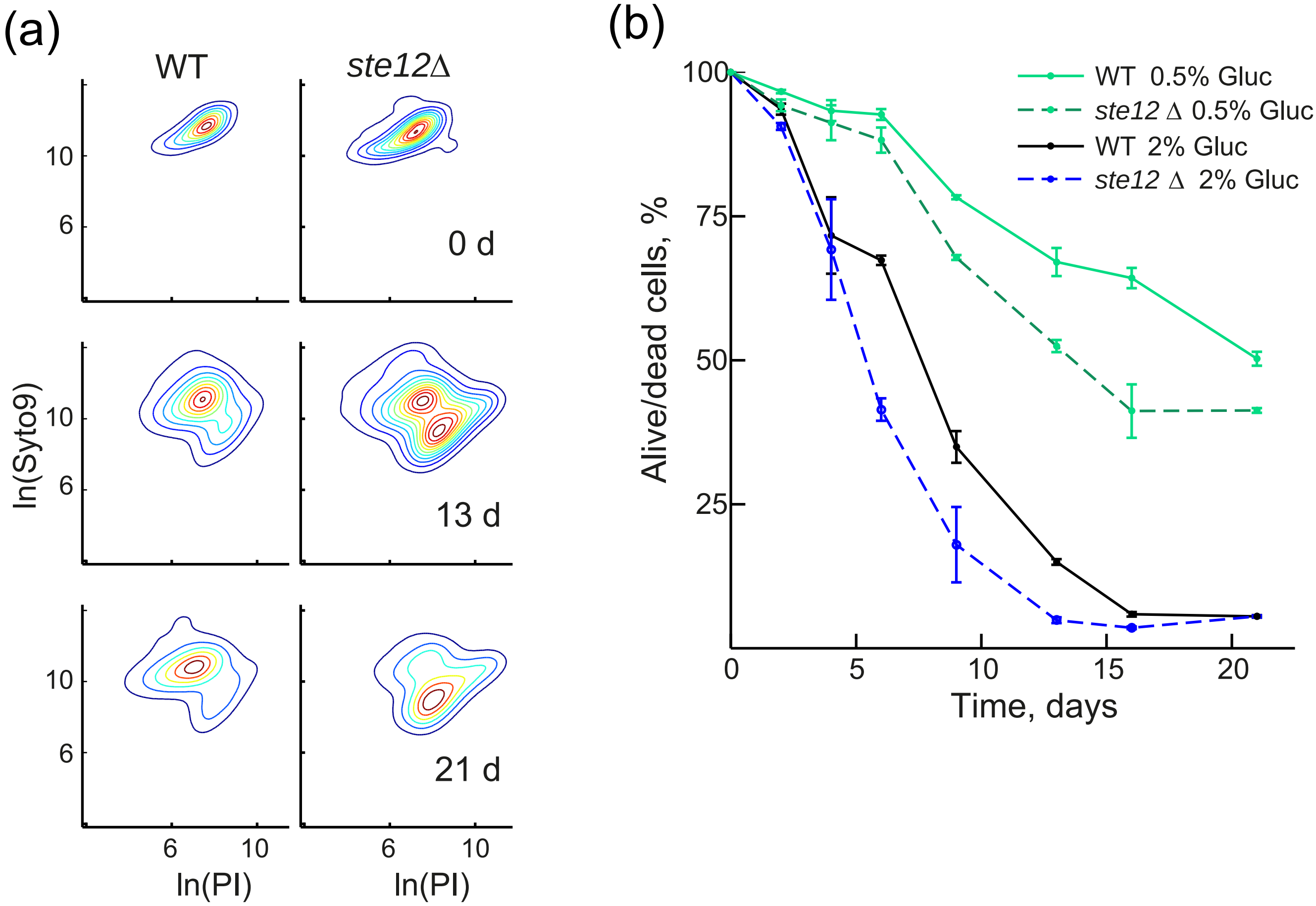

Figure S7. Alive/dead cell staining confirms a role of Ste12 in lifespan extension by dietary restriction. (**a**) Contour plots showing WT and *ste12*Δ populations of dead and alive cells under SC 0.5% glucose; fluorescence of Syto9 (alive cells) and propidium iodide (PI, dead cells) is shown in the vertical and horizontal axes, respectively, across time in stationary phase. (**b**) Alive/dead assay plotted to show the fraction of alive/dead cells in populations of a WT (solid lines) or *ste12*Δ (dashed lines) strains aged under 2% or 0.5% glucose. The fraction of alive cells was calculated by dividing Syto9 (alive) stained cells by the total number of events at each time-point. Cells were grown in non-aerated 96-deepwell plates, a sample was taken in the days indicated in the plot for flow cytometry. Replicates were aged in parallel in the same plate for the WT and mutant strains. Error bars are the S.E.M. (*n*=3).
