# Supplementary material for: Genomewide mechanisms of chronological longevity by dietary restriction in budding yeast
Source: Aging Cell. 2018 Mar 25;17(3):e12749. doi: 10.1111/acel.12749 (PMC5946063; doi:10.1111/acel.12749)
Supplement: Supplementary file 8 [file ACEL-17-e12749-s008.pdf]

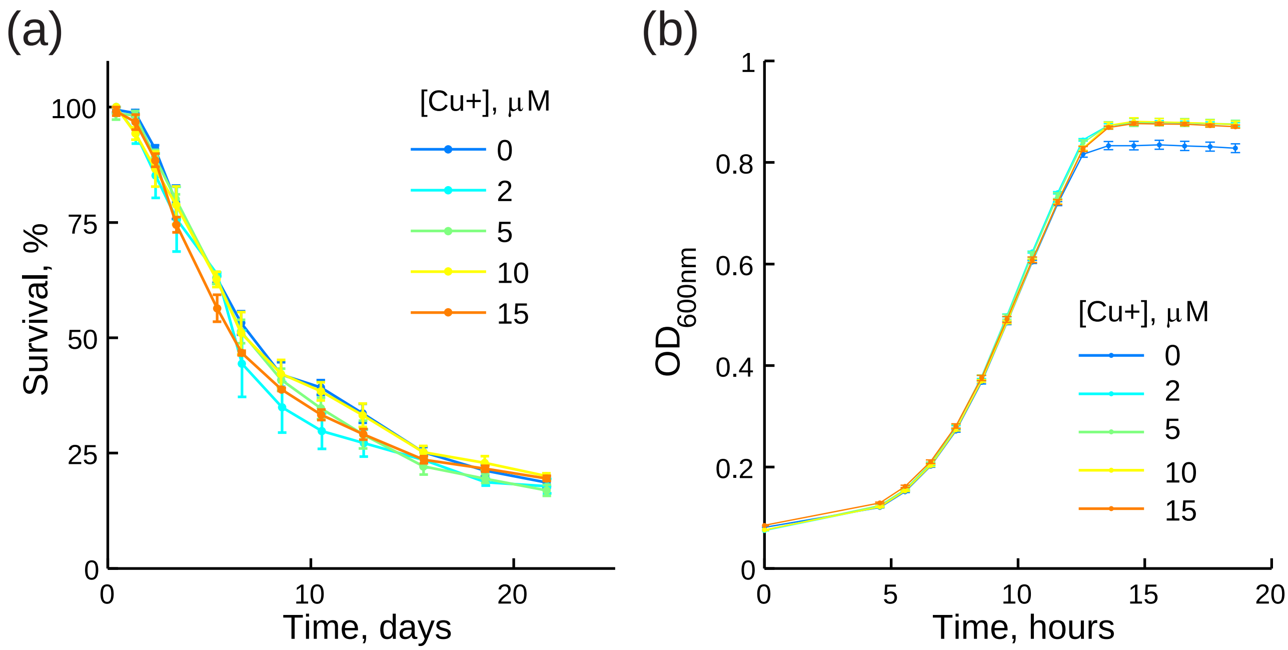

Figure S8. WT cells are not affected in their survival nor growth by copper treatment. (**a**) Survival curves of a WT strain treated with increasing concentrations of copper sulfate. Different concentrations of copper in the WT were aged in a single deepwell plate with no aeration (*n*=5) (**b**) Plot shows growth curves of WT strain treated with increasing concentrations of copper sulfate. Error bars are the S.E.M. (*n*=8).
