# Supplementary material for: Genomewide mechanisms of chronological longevity by dietary restriction in budding yeast
Source: Aging Cell. 2018 Mar 25;17(3):e12749. doi: 10.1111/acel.12749 (PMC5946063; doi:10.1111/acel.12749)
Supplement: Supplementary file 9 [file ACEL-17-e12749-s009.pdf]

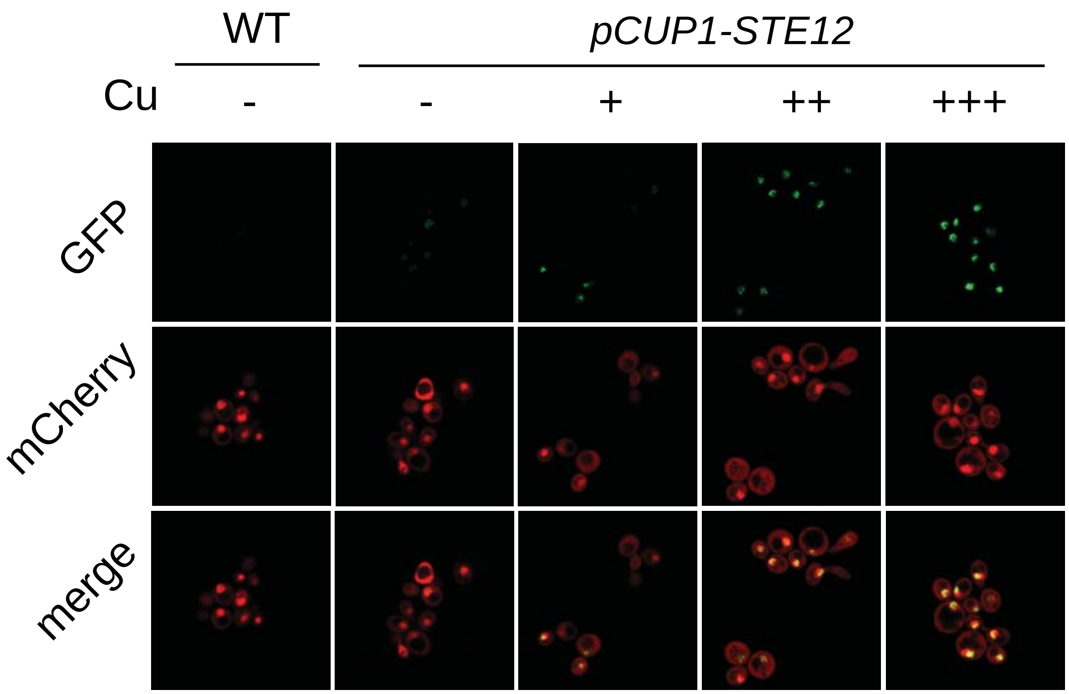

Figure S9. Confocal microscopy of *STE12* over-expression cells. Micrographs of WT and *pCUP1::GFP-STE12* strains with GFP, mCherry (constitutive), and merge filters, showing untreated (-) or induced cells with 2µM (+), 5µM (++), or 15 µM (+++) copper sulfate.
