# Supplementary material for: Genomewide mechanisms of chronological longevity by dietary restriction in budding yeast
Source: Aging Cell. 2018 Mar 25;17(3):e12749. doi: 10.1111/acel.12749 (PMC5946063; doi:10.1111/acel.12749)
Supplement: Supplementary file 10 [file ACEL-17-e12749-s010.pdf]

Supporting Table S1. Chronological lifespan assays, aging media, and strains used in this study

CLS assay	Experimental setup	Media and recipes [a,b]		Strain genotypes tested
Genome screen, as (Garay et al. 2014)	Co-culture of WT and mutant strains grown and aged in 96-deepwells with 700 µl medium/well, incubated with low aeration in robotic platform at 30 °C	SC Gln	2% glucose, 0.17% YNB without amino acids and ammonium sulfate, 0.07% amino acid mix, 25 mM glutamine	*MATa xxxΔ::kanMX4 PDC1-mCherry-CaURA3MX4 can1Δ::STE2pr-SpHIS5 lyp1Δ his3Δ1 ura3Δ0 leu2Δ1::LEU2*
		SC GABA	2% glucose, 0.17% YNB without amino acids and ammonium sulfate, 0.07% amino acid mix, 25 mM GABA	<u>WT reference</u><br>*MATa his3Δ1::kanMX4 PDC1-Cerulean-CaURA3MX4 can1Δ::STE2pr-SpHIS5 lyp1Δ ura3Δ0 leu2Δ1::LEU2*
Small-scale assay, modified from (Murakami et al. 2008)	Single-strain cultures grown and aged in 96-deepwells with 700 µl medium/well, incubated with low aeration in robotic platform at 30 °C	SC Gln	2% glucose, 0.17% YNB without amino acids and ammonium sulfate, 0.07% amino acid mix, 25 mM glutamine	<u>Genome screen confirmation</u><br>*MATa xxxΔ::KANMX4 PDC1-mCherry-CaURA3MX4 can1Δ::STE2pr-SpHIS5 lyp1Δ his3Δ1 ura3Δ0 leu2Δ1::LEU2*<br>(*SpHIS5* is expressed in *MATa*)
		SC GABA	2% glucose, 0.17% YNB without amino acids and ammonium sulfate, 0.07% amino acid mix, 25 mM GABA	
		SC 2% Gluc	2% glucose, 0.67% YNB without amino acids, 0.2% amino acid mix (ammonium is the nitrogen source)	<u>De novo gene deletions</u><br>*MATα xxxΔ::NATMX6 PDC1-mCherry-CaURA3MX4 can1Δ::STE2pr-SpHIS5 lyp1Δ his3Δ1 ura3Δ0 leu2Δ1::LEU2*<br>(*SpHIS5* is not expressed in *MATα*)
		SC 0.5% Gluc	0.5% glucose, 0.67% YNB without amino acids, 0.2% amino acid mix (ammonium is the nitrogen source)	
Standard CLS assay, as (Hu et al. 2013)	Single cultures grown and aged in 50 ml glass tubes with aluminum foil cap and 10 ml of medium, incubated with high aeration at 200 rpm in orbital shaker at 30 °C	SC 2% Gluc	2% glucose, 0.67% YNB without amino acids, 0.2% amino acid mix (ammonium is the nitrogen source)	*MATα xxxΔ::NATMX6 PDC1-mCherry-CaURA3MX4 can1Δ::STE2pr-SpHIS5 lyp1Δ his3Δ1 ura3Δ0 leu2Δ1::LEU2*<br>(*SpHIS5* is not expressed in *MATα*)
		SC 0.5% Gluc	0.5% glucose, 0.67% YNB without amino acids, 0.2% amino acid mix (ammonium is the nitrogen source)	

a. Composition of the amino-acid supplement mix is based on the CSHL manual is provided in Supporting Workbook 1.

b. None of the aging media were buffered.
